# Macroscopic coherent structures in a stochastic neural network: from interface dynamics to coarse-grained bifurcation analysis

**DOI:** 10.1007/s00285-016-1070-9

**Published:** 2017-02-01

**Authors:** Daniele Avitable, Kyle C. A. Wedgwood

**Affiliations:** 1 Centre for Mathematical Medicine and Biology, School of Mathematical Sciences, University of Nottingham, Nottingham, NG2 7RD UK; 2 Centre for Biomedical Modelling and Analysis, University of Exeter, Living Systems Institute, Stocker Road, Exeter, EX4 4QD UK

**Keywords:** Multiple scale analysis, Mathematical neuroscience, Refractoriness, Spatio-temporal patterns, Equation-free modelling, Markov chains, 37N25, 34E13, 35B34

## Abstract

We study coarse pattern formation in a cellular automaton modelling a spatially-extended stochastic neural network. The model, originally proposed by Gong and Robinson (Phys Rev E 85(5):055,101(R), 2012), is known to support stationary and travelling bumps of localised activity. We pose the model on a ring and study the existence and stability of these patterns in various limits using a combination of analytical and numerical techniques. In a purely deterministic version of the model, posed on a continuum, we construct bumps and travelling waves analytically using standard interface methods from neural field theory. In a stochastic version with Heaviside firing rate, we construct approximate analytical probability mass functions associated with bumps and travelling waves. In the full stochastic model posed on a discrete lattice, where a coarse analytic description is unavailable, we compute patterns and their linear stability using equation-free methods. The lifting procedure used in the coarse time-stepper is informed by the analysis in the deterministic and stochastic limits. In all settings, we identify the synaptic profile as a mesoscopic variable, and the width of the corresponding activity set as a macroscopic variable. Stationary and travelling bumps have similar meso- and macroscopic profiles, but different microscopic structure, hence we propose lifting operators which use microscopic motifs to disambiguate them. We provide numerical evidence that waves are supported by a combination of high synaptic gain and long refractory times, while meandering bumps are elicited by short refractory times.

## Introduction

In the past decades, single-neuron recordings have been complemented by multineuronal experimental techniques, which have provided quantitative evidence that the cells forming the nervous systems are coupled both structurally (Braitenberg and Schüz 1998) and functionally (for a recent review, see Yuste (2015) and references therein). An important question in neuroscience concerns the relationship between electrical activity at the level of individual neurons and the emerging spatio-temporal coherent structures observed experimentally using local field potential recordings (Einevoll et al. 2013), functional magnetic resonance imaging (Heuvel and Hulshoff Pol 2010) and electroencephalography (Nunez and Srinivasan 2006).

There exist a wide variety of models describing activity at the level of an individual neuron (Izhikevich 2007; Ermentrout and Terman 2010), and major research efforts in theoretical and computational neuroscience are directed towards coupling neurons in large-dimensional neural networks, whose behaviour is studied mainly via direct numerical simulations (Izhikevich and Edelman 2008; Fairhall and Sompolinsky 2014).

A complementary approach, dating back to Wilson and Cowan (1972, 1973) and Amari (1975, 1977), foregoes describing activity at the single neuron level by representing averaged activity across populations of neurons. These *neural field models* are nonlocal, spatially-extended, excitable pattern-forming systems (Ermentrout 1998) which are often analytically tractable and support several coherent structures such as localised radially-symmetric states (Werner and Richter 2001; Laing et al. 2002; Laing and Troy 2003; Bressloff and Kilpatrick 2011; Faye et al. 2013), localised patches (Laing and Troy 2003; Rankin et al. 2014; Avitabile and Schmidt 2015), patterns on lattices with various symmetries (Ermentrout and Cowan 1979; Bressloff et al. 2001), travelling bumps and fronts (Ermentrout and McLeod 1993; Bressloff 2014), rings (Owen et al. 2007; Coombes et al. 2012), breathers (Folias and Bressloff 2004, 2005; Folias and Ermentrout 2012), target patterns (Coombes et al. 2013), spiral waves (Laing 2005) and lurching waves (Golomb and Ermentrout 1999; Osan and Ermentrout 2001; Wasylenko et al. 2010) [for comprehensive reviews, we refer the reader to Bressloff 2012, 2014].

Recent studies have analysed neural fields with additive noise (Hutt et al. 2008; Faugeras et al. 2009; Kuehn and Riedler 2014), multiplicative noise (Bressloff and Webber 2012), or noisy firing thresholds (Brackley and Turner 2007), albeit these models are still mostly phenomenological. Even though several papers derive continuum neural fields from microscopic models of coupled neurons (Jirsa and Haken 1997; Bressloff 2009, 2010; Baladron et al. 2012), the development of a rigorous theory of multi-scale brain models is an active area of research.

Numerical studies of networks based on realistic neural biophysical models rely almost entirely on brute-force Monte Carlo simulations (for a very recent, remarkable example, we refer the reader to (Markram et al. 2015)). With this *direct numerical simulation* approach, the stochastic evolution of each neuron in the network is monitored, resulting in huge computational costs, both in terms of computing time and memory. From this point of view, multi-scale numerical techniques for neural networks present interesting open problems.

When few clusters of neurons with similar properties form in the network, a significant reduction in computational costs can be obtained by population density methods (Omurtag et al. 2000; Haskell et al. 2001), which evolve probability density functions of neural subpopulations, as opposed to single neuron trajectories. This coarse-graining technique is particularly effective when the underlying microscopic neuronal model has a low-dimensional state space (such as the leaky integrate-and-fire model) but its performance degrades for more realistic biophysical models. Developments of the population density method involve analytically derived moment closure approximations (Cai et al. 2004; Ly and Tranchina 2007). Both Monte Carlo simulations and population density methods give access only to stable asymptotic states, which may form only after long-transient simulations.

An alternative approach is offered by *equation-free* (Kevrekidis et al. 2003; Kevrekidis and Samaey 2009) and *heterogeneous multiscale* methods (Weinan and Engquist 2003; Weinan et al. 2007), which implement multiple-scale simulations using an on-the-fly numerical closure approximations. Equation-free methods, in particular, are of interest in computational neuroscience as they accelerate macroscopic simulations and allow the computation of unstable macroscopic states. In addition, with equation-free methods, it is possible to perform coarse-grained bifurcation analysis using standard numerical bifurcation techniques for time-steppers (Tuckerman and Barkley 2000).

The equation-free framework (Kevrekidis et al. 2003; Kevrekidis and Samaey 2009) assumes the existence of a closed coarse model in terms of a few macroscopic state variables. The model closure is enforced numerically, rather than analytically, using a *coarse time-stepper*: a computational procedure which takes advantage of knowledge of the microscopic dynamics to time-step an approximated macroscopic evolution equation. A single coarse time step from time $t_0$ to time $t_1$ is composed of three stages: (i) *lifting*, that is, the creation of microscopic initial conditions that are compatible with the macroscopic states at time $t_0$; (ii) *evolution*, the use of independent realisations of the microscopic model over a time interval $[t_0, t_1]$; (iii) *restriction*, that is, the estimation of the macroscopic state at time $t_1$ using the realisations of the microscopic model.

While equation-free methods have been employed in various contexts (see Kevrekidis and Samaey (2009) and references therein) and in particular in neuroscience applications (Laing 2006; Laing et al. 2007, 2010; Spiliotis and Siettos 2011, 2012; Laing and Kevrekidis 2015), there are still open questions, mainly related to how noise propagates through the coarse time stepper. A key aspect of every equation-free implementation is the lifting step. The underlying lifting operator, which maps a macroscopic state to a set of microscopic states, is generally non-unique, and lifting choices have a considerable impact on the convergence properties of the resulting numerical scheme (Avitabile et al. 2014). Even though the choice of coarse variables can be automatised using data-mining techniques, as shown in several papers by Laing, Kevrekidis and co-workers (Laing 2006; Laing et al. 2007, 2010), the lifting step is inherently problem dependent.

The present paper explores the possibility of using techniques from neural field theory to inform the coarse-grained bifurcation analysis of discrete neural networks. A successful strategy in analysing neural fields is to replace the models’ sigmoidal firing rate functions with Heaviside distributions (Bressloff 2012, 2014). Using this strategy, it is possible to relate macroscopic observables, such as bump widths or wave speeds, to biophysical parameters, such as firing rate thresholds. Under this hypothesis, a macroscopic variable suggests itself, as the state of the system can be constructed entirely via the loci of points in physical space where the neural activity attains the firing-rate threshold value. In addition, there exists a closed (albeit implicit) evolution equation for such interfaces (Coombes et al. 2012).

In this study, we show how the insight gained in the Heaviside limit may be used to perform coarse-grained bifurcation analysis of neural networks, even in cases where the network does not evolve according to an integro-differential equation. As an illustrative example, we consider a spatially-extended neural network in the form of a discrete time Markov chain with discrete ternary state space, posed on a lattice. The model is an existing cellular automaton proposed by Gong and Robinson (2012), and it has been related to neuroscience in the context of relevant spatio-temporal activity patterns that are observed in cortical tissue. In spite of its simplicity, the model possesses sufficient complexity to support rich dynamical behaviour akin to that produced by neural fields. In particular, it explicitly includes refractoriness and is one of the simplest models capable of generating propagating activity in the form of travelling waves. An important feature of this model is that the microscopic transition probabilities depend on the local properties of the tissue, as well as on the global synaptic profile across the network. The latter has a convolution structure typical of neural field models, which we exploit to use interface dynamics and define a suitable lifting strategy.

We initially study the model in simplifying limits in which an analytical (or semi-analytical) treatment is possible. In these cases, we construct bump and wave solutions and compute their stability. This analysis follows the standard Amari framework, but is here applied directly to the cellular automaton. We then derive the corresponding lifting operators, which highlight a critical importance of the microscopic structure of solutions: one of the main results of our analysis is that, since macroscopic stationary and travelling bumps coexist and have nearly identical macroscopic profiles, a standard lifting is unable to distinguish between them, thereby preventing coarse numerical continuation. These solutions, however, possess different microstructures, which are captured by our analysis and subsequently by our lifting operators. This allows us to compute separate solution branches, in which we vary several model parameters, including those associated with the noise processes.

The manuscript is arranged as follows: In Sect. 2 we outline the model. In Sect. 3, we simulate the model and identify the macroscopic profiles in which we are interested, together with the coarse variables that describe them. In Sect. 4, we define a deterministic version of the full model and lay down the framework for analysing it. In Sects. 5 and 6, we respectively construct bump and wave solutions under the deterministic approximation and compute the stability of these solutions. In Sect. 7, we define and construct travelling waves relaxing the deterministic limit. In Sects. 8.1 and 8.2, we provide the lifting steps for use in the equation-free algorithm for the bump and wave respectively. In Sect. 9, we briefly outline the continuation algorithm and in Sect. 10, we show the results of applying this continuation to our system. Finally, in Sect. 11, we make some concluding remarks.

## Model description

### State variables for continuum and discrete tissues

In this section, we present a modification of a model originally proposed by Gong and Robinson (2012). We consider a one-dimensional neural tissue $\mathbb {X}\subset \mathbb {R}$. At each discrete time step $t \in \mathbb {Z}$, a neuron at position $x \in \mathbb {X}$ may be in one of three states: a refractory state (henceforth denoted as $-1$), a quiescent state (0) or a spiking state (1). Our state variable is thus a function $u :\mathbb {X}\times \mathbb {Z}\rightarrow \mathbb {U}$, where $\mathbb {U}=\{-1,0,1\}$. We pose the model on a continuum tissue $\mathbb {S}= \mathbb {R}/2L\mathbb {Z}$ or on a discrete tissue featuring $N+1$ evenly spaced neurons,

$$\begin{aligned} \mathbb {S}_N = \{x_i\}_{i=0}^N, \qquad x_i = -L + i2L /N \in [-L,L]. \end{aligned}$$

We will often alternate between the discrete and the continuum setting, hence we will use a unified notation for these cases. We use the symbol $\mathbb {X}$ to refer to either $\mathbb {S}$ or $\mathbb {S}_N$, depending on the context. Also, we use $u({\,\cdot \,},t)$ to indicate the state variable in both the discrete and the continuum case: $u({\,\cdot \,},t)$ will denote a step function defined on $\mathbb {S}$ in the continuum case and a vector in $\mathbb {U}^N$ with components $u(x_i,t)$ in the discrete case. Similarly, we write $\int _\mathbb {X}u(x) \,\mathrm {d}x$ to indicate $\int _\mathbb {S}u(x) \, \mathrm {d}x$ or $2L/N \sum _{j=0}^N u(x_j)$.

### Model definition

**Fig. 1:**
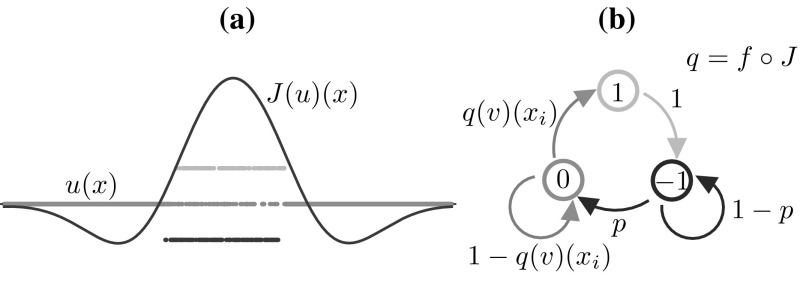
**a** Example of a state $u(x) \in \mathbb {U}^N$ and corresponding synaptic profile $J(u)(x) \in \mathbb {R}^N$ in a stochastic network of 1024 neurons. **b** Schematic of the transition kernel for the network (see also Eqs. (5)–(7)). The conditional probability of the *local* variable $u(x_i,t+1)$ depends on the *global* state of the network at time *t*, via the function $q = f \circ J$, as seen in (7)

We use the term *stochastic model* when the Markov chain model described below is posed on $\mathbb {S}_N$. An example of a state supported by the stochastic model is given in Fig. 1a.

In the model, neurons are coupled via a translation-invariant synaptic kernel, that is, we assume the connection strength between two neurons to be dependent on their mutual Euclidean distance. In particular, we prescribe that short range connections are excitatory, whilst long-range connections are inhibitory. To model this coupling, we use a standard Mexican hat function,

1 $$\begin{aligned} w :\mathbb {X}\rightarrow \mathbb {R}, \qquad x \mapsto A_1 \sqrt{B_1/L} \exp (-4 B_1 x^2) - A_2 \sqrt{B_2/L} \exp (-4 B_2 x^2), \end{aligned}$$

and denote by *W* its periodic extension.

In order to describe the dynamics of the model, it is useful to partition the tissue $\mathbb {X}$ into the 3 pullback sets

2 $$\begin{aligned} X^u_k(t) = \{ x \in \mathbb {X}:u(x,t) = k \}, \quad k \in \mathbb {U}, \quad t \in \mathbb {Z}, \end{aligned}$$

so that we can write, for instance, $X^u_1(t)$ to denote the set of neurons that are firing at time *t* (and similarly for $X^u_{-1}$ and $X^u_0$). Where it is unambiguous, we shall simply write $X_k$ or $X_k(t)$ in place of $X^u_k(t)$.

The synaptic input to a cell at position $x_i$ is given by a weighted sum of inputs from all firing cells. Using the synaptic kernel (1) and the partition (2), the synaptic input is then modelled as

3 $$\begin{aligned} J :\mathbb {X}\times \mathbb {Z}\rightarrow \mathbb {R}, \qquad (x,t) \mapsto \kappa \int _{\mathbb {X}} W(x - y) {{\mathbbm {1}}}_{X_1(t)}(y) \, \mathrm {d}y = \kappa \int _{X_1(t)} W(x - y) \, dy, \end{aligned}$$

where $\kappa \in \mathbb {R}_+$ is the synaptic gain, which is common for all neurons and ${{\mathbbm {1}}}_X$ is the indicator function of a set *X*.

#### Remark 1

(Dependence of *J* on *u*) Since $X_1$ depends on the state variable *u*, so does the synaptic input (3). Where necessary, we will write *J*(*u*)(*x*, *t*) to highlight the dependence on *u*. We refer the reader to Fig. 1 for a concrete example of synaptic profile.

The firing probability associated to a quiescent neuron is linked to the synaptic input via the firing rate function

4 $$\begin{aligned} f :\mathbb {R}\rightarrow \mathbb {R}, \qquad I \mapsto \frac{1}{1+ \exp [-\beta (I - h)]}, \end{aligned}$$

whose steepness and threshold are denoted by the positive real numbers $\beta$ and *h*, respectively. We are now ready to describe the evolution of the stochastic model, which is a discrete-time Markov process with finite state space $\mathbb {U}^N$ and transition probabilities specified as follows: for each $x_i \in \mathbb {S}_N$ and $t \in \mathbb {Z}$

5 $$\begin{aligned}&{{\mathrm{Pr}}}\big [ u(x_i,t+1) = -1 \big | u(x,t) = v(x) \big ] = {\left\{ \begin{array}{ll} 1-p &{} \text {if }v(x_i)=-1, \\ 1 &{} \text {if }v(x_i)= 1, \\ 0 &{} \text {otherwise,} \end{array}\right. } \end{aligned}$$

6 $$\begin{aligned}&{{\mathrm{Pr}}}\big [ u(x_i,t+1) = 0 \big | u(x,t) = v(x) \big ] = {\left\{ \begin{array}{ll} p &{} \text {if }v(x_i)=-1, \\ 1-f(J(v))(x_i) &{} \text {if }v(x_i)= 0, \\ 0 &{} \text {otherwise,} \end{array}\right. } \end{aligned}$$

7 $$\begin{aligned}&{{\mathrm{Pr}}}\big [ u(x_i,t+1) = 1 \big | u(x,t) = v(x) \big ] = {\left\{ \begin{array}{ll} f(J(v))(x_i) &{} \text {if }v(x_i)= 0, \\ 0 &{} \text {otherwise,} \end{array}\right. } \end{aligned}$$

where $p \in (0,1]$. We give a schematic representation of the transitions of each neuron in the network in Fig. 1b. We remark that conditional probability of the *local* variable $u(x_i,t+1)$ depends on the *global* state of the network at time *t*, via the function $f \circ J$.

The model described by (1)–(7), complemented by initial conditions, defines a stochastic evolution map that we will formally denote as

8 $$\begin{aligned} u(x,t+1) = \varphi (u(x,t); \gamma ), \end{aligned}$$

where $\gamma =(\kappa ,\beta ,h,p,A_1,A_2,B_1,B_2)$ is a vector of control parameters.

#### Remark 2

**Fig. 2:**
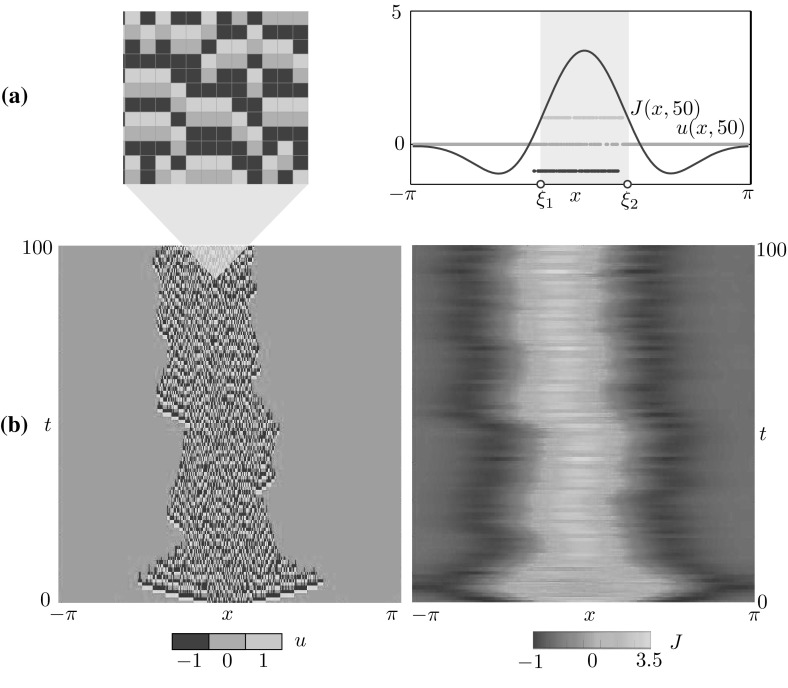
Bump obtained via time simulation of the stochastic model for $(x,t) \in [-\pi ,\pi ] \times [0,100]$. **a** The microscopic state *u*(*x*, *t*) (*left*) attains the discrete values $-1$ (*blue*), 0 (*green*) and 1 (*yellow*). The corresponding synaptic profile *J*(*x*, *t*) is a continuous function. A comparison between *J*(*x*, 50) and *u*(*x*, 50) is reported on the right panel, where we also mark the interval $[\xi _1, \xi _2]$ where *J* is above the firing threshold *h*. **b** Space-time plots of *u* and *J*. Parameters as in Table 1

**Fig. 3:**
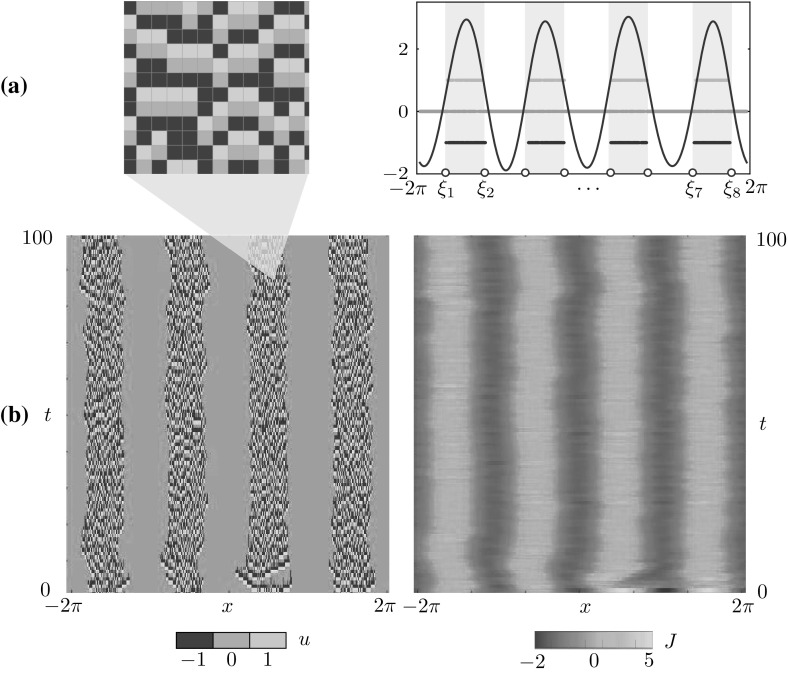
Multiple bump solution obtained via time simulation of the stochastic model for $(x,t) \in [-2\pi ,2\pi ] \times [0,100]$. **a** The microscopic state *u*(*x*, *t*) in the active region (*left*) is similar to the one found for the single bump (see Fig. 2a). A comparison between *J*(*x*, 50) and *u*(*x*, 50) is reported on the right panel, where we also mark the intervals $[\xi _1, \xi _2], \ldots , [\xi _7,\xi _8]$ where *J* is above the firing threshold *h*. **b** Space-time plots of *u* and *J*. Parameters are as in Table 1

**Fig. 4:**
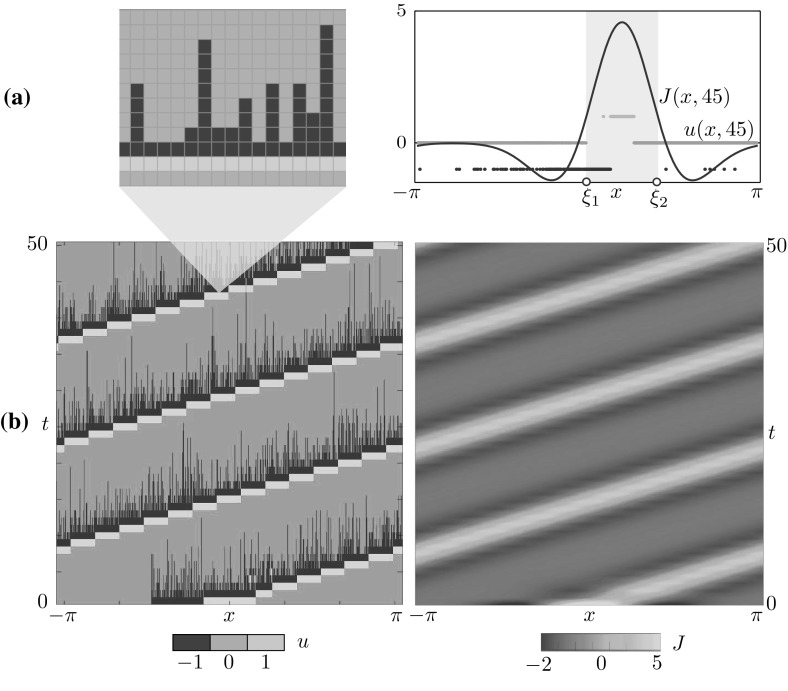
Travelling wave obtained via time simulation of the stochastic model for $(x,t) \in [-\pi ,\pi ] \times [0,50]$. **a** The microscopic state *u*(*x*, *t*) (left) has a characteristic microstructure, which is also visible on the right panel, where we compare *J*(*x*, 45) and *u*(*x*, 45). As in the other cases, we mark the interval $[\xi _1, \xi _2]$ where *J* is above the firing threshold *h*. **b** Space-time plots of *u* and *J*. Parameters are as in Table 1

(Microscopic, mesoscopic and macroscopic descriptions) We will henceforth use the terms “microscopic”, “mesoscopic” and “macroscopic” to refer to different state variables or model descriptions. Examples of these three state variables appear together in Figs. 2, 3 and 4 in Sect. 3, and we introduce them briefly here:

**Microscopic level**: Model (8) will be referred to as microscopic model and its solutions at a fixed time *t* as microscopic states. We will use these terms also when $p=1$ and $\beta \rightarrow \infty$, that is, when the evolution Eq. (8) is deterministic.
**Mesoscopic level**: In Remark 1, we associated to each microscopic state *u* a corresponding synaptic profile *J*, which is smooth, even when the tissue is discrete. We will not seek an evolution equation for the variable *J*, as the corresponding dynamical system would not reprent a reduction of the microscopic one. However, we will use *J* to bridge between the microscopic and macroscopic model descriptions; we therefore refer to *J* as a mesoscopic variable (or mesoscopic state).
**Macroscopic level**: Much of the present paper aims to show that, for the model under consideration, there exists a high-level model description, in the spirit of interfacial dynamics for neural fields (Bressloff 2012; Coombes et al. 2012; Bressloff 2014). The state variables for this level are points on the tissue where *J*(*u*)(*x*, *t*) attains the firing rate threshold *h*. We will denote these threshold crossings as $\{\xi _i(t)\}$ and we will discuss (reduced) evolution equations in terms of $\xi _i(t)$. The variables $\{ \xi (t) \}$ are therefore referred to as macroscopic variables and the corresponding evolution equations as the macroscopic model.

## Microscopic states observed via direct simulation

In this section, we introduce a few coherent states supported by the stochastic model. The main aim of the section is to show examples of bumps, multiple bumps and travelling waves, whose existence and stability properties will be studied in the following sections. In addition, we give a first characterisation of the macroscopic variables of interest and link them to the microscopic structure observed numerically.

### Bumps

In a suitable region of parameter space, the microscopic model supports bump solutions (Qi and Gong 2015) in which the microscopic variable *u*(*x*, *t*) is nonzero only in a localised region of the tissue. In this *active* region, neurons attain all values in $\mathbb {U}$. In Fig. 2, we show a time simulation of the microscopic model with $N=1024$ neurons. At each time *t*, neurons are in the refractory (blue), quiescent (green) or spiking (yellow) state. We prescribe the initial condition by setting $u(x_i,0)=0$ outside of a localised region, in which $u(x_i,0)$ are sampled randomly from $\mathbb {U}$. After a short transient, a stochastic *microscopic* bump is formed. As expected due to the stochastic nature of the system (Kilpatrick and Ermentrout 2013), the active region wanders while remaining localised. A space-time section of the active region reveals a characteristic random microstructure (see Fig. 2a).

By plotting *J*(*x*, *t*), we see that the active region is well approximated by the portion of the tissue $X_\ge = [\xi _1, \xi _2]$ where *J* lies above the threshold *h*. A quantitative comparison between *J*(*x*, 50) and *u*(*x*, 50) is made in Fig. 2a. We interpret *J* as a *mesoscopic* variable associated with the bump, and $\xi _1$ and $\xi _2$ as corresponding *macroscopic* variables (see also Remark 2).

### Multiple-bumps solutions

Solutions with multiple bumps are also observed by direct simulation, as shown in Fig. 3. The microstructure of these patterns resembles the one found in the single bump case (see Fig. 3a). At the mesoscopic level, the set for which *J* lies above the threshold *h* is now a union of disjoint intervals $[\xi _1,\xi _2], \ldots , [\xi _7,\xi _8]$. The number of bumps of the pattern depends on the width of the tissue; the experiment of Fig. 3 is carried out on a domain twice as large as that of Fig. 2. The examples of bump and multiple-bump solutions reported in these figures are obtained for different values of the main control parameter $\kappa$ (see Table 1), however, these states coexist in a suitable region of parameter space, as will be shown below.

### Travelling waves

Further simulation shows that the model also supports coherent states in the form of stochastic travelling waves. In two spatial dimensions, the system is known to support travelling spots (Gong and Robinson 2012; Qi and Gong 2015). In Fig. 4, we show a time simulation of the stochastic model with initial condition

$$\begin{aligned} u(x,0) = \sum _{k \in \mathbb {U}} k {\mathbbm {1}}_{X_k}(x) \quad \text {with partition} \quad \begin{aligned} X_{-1}&= [-1.5,-0.5), \\ X_{0}&= [-\pi ,-1.5)\cup [0.5,\pi ), \\ X_{1}&= [-0.5,0.5). \end{aligned} \end{aligned}$$

In passing, we note that the state of the network at each discrete time *t* is defined entirely by the partition $\{X_k\}$ of the tissue; we shall often use this characterisation in the reminder of the paper.

**Table 1:** Parameter values for which the stochastic model supports a bump (Fig. 2), a multiple-bump solution (Fig. 3) and a travelling wave (Fig. 4).

Experiment	$\kappa$	$\beta$	*h*	*p*	$A_1$	$A_2$	$B_1$	$B_2$	*N*	*L*
Bump	30	5	0.9	0.7	5.25	5	0.2	0.3	1024	$\pi$
Multiple bump	60	5	0.9	0.7	5.25	5	0.2	0.3	2058	$2\pi$
Travelling wave	30	$\infty$	1.0	0.4	5.25	5	0.2	0.3	1024	$\pi$

The value $\infty$ for the parameter $\beta$ indicates that a Heaviside firing rate has been used in place of the sigmoidal function (4)

**Fig. 5:**
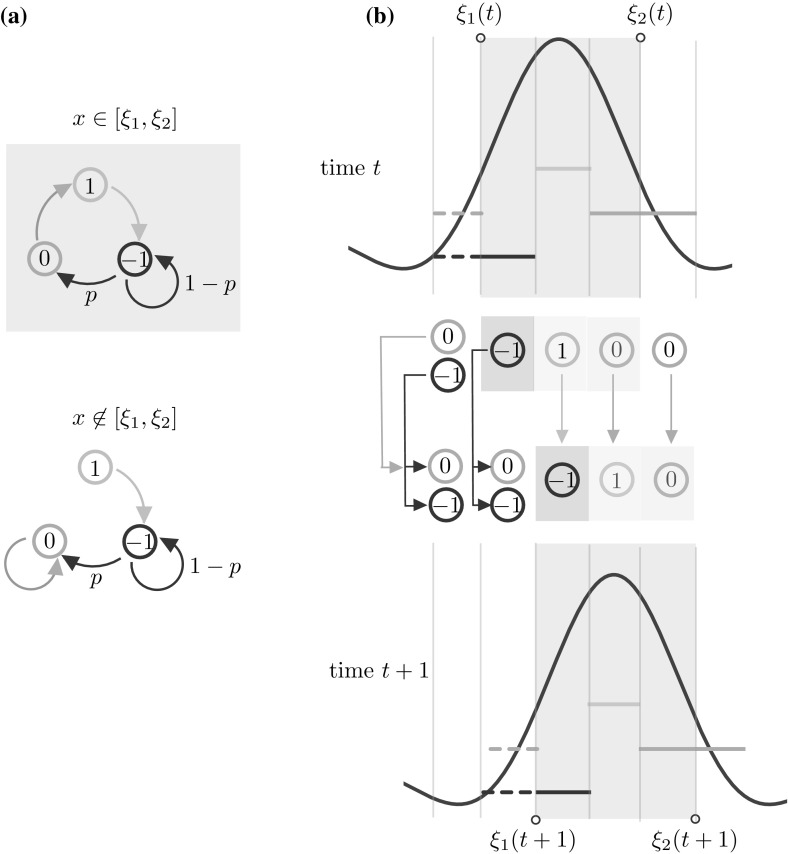
Schematic of the advection mechanism for the travelling wave state. Shaded areas pertain to the active region $[\xi _1(t),\xi _2(t)]$, non-shaded areas to the inactive region $\mathbb {X}{\setminus } [\xi _1(t),\xi _2(t)]$. **a** In the active (inactive) region, $q_i = f(J(u))(x_i) \approx 1$ ($q_i \approx 0$), hence the transition kernel (5)–(7) can be simplified as shown. **b** At time *t* the travelling wave has a profile similar to the one in Fig. 4, which we represent in the proximity of the active region. We depict 5 intervals of equal width, 3 of which form a partition of $[\xi _1(t),\xi _2(t)]$. Each interval is mapped to another interval at time $t+1$, following the transition rules sketched in (**a**). In one discrete step, the wave progresses with positive speed: so that $J(x,t+1)$ is a translation of *J*(*x*, *t*)

In the direct simulation of Fig. 4, the active region moves to the right and, after just 4 iterations, a travelling wave emerges. The microscopic variable, *u*(*x*, *t*), displays stochastic fluctuations which disappear at the level of the mesoscopic variable, *J*(*x*, *t*), giving rise to a seemingly deterministic travelling wave. A closer inspection (Fig. 4a) reveals that the state can still be described in terms of the active region $[\xi _1, \xi _2]$ where *J* is above *h*. However, the travelling wave has a different microstructure with respect to the bump. Proceeding from right to left, we observe:

1. A region of the tissue ahead of the wave, $x \in (\xi _2,\pi )$, where the neurons are in the quiescent state 0 with high probability.
2. An active region $x \in [\xi _1,\xi _2]$, split in three subintervals, each of approximate width $(\xi _2 - \xi _1)/3$, where *u* attains with high probability the values 0, 1 and $-1$ respectively.
3. A region at the back of the wave, $x \in [-\pi ,\xi _1)$, where neurons are either quiescent or refractory. We note that $u=0$ with high probability as $x \rightarrow -\pi$ whereas, as $x \rightarrow \xi _1$, neurons are increasingly likely to be refractory, with $u=-1$.

A further observation of the space-time plot of *u* in Fig. 4b reveals a remarkably simple advection mechanism of the travelling wave, which can be fully understood in terms of the transition kernel of Fig. 1b upon noticing that, for sufficiently large $\beta$, $q_i = f(J(u))(x_i) \approx 0$ everywhere except in the active region, where $q_i \approx 1$. In Fig. 5, we show how the transition kernel simplifies inside and outside the active region and provide a schematic of the advection mechanism. For an idealised travelling wave profile at time *t*, we depict 3 subintervals partitioning the active region (shaded), together with 2 adjacent intervals outside the active region. Each interval is then mapped to another interval, following the simplified transition rules sketched in Fig. 5a:

1. At the front of the wave, to the right of $\xi _2(t)$, neurons in the quiescent state 0 remain at 0 (rules for $x \not \in [\xi _1,\xi _2]$).
2. Inside the active region, to the left of $\xi _2(t)$, we follow the rules for $x \in [\xi _1,\xi _2]$ in a clockwise manner: neurons in the quiescent state 0 spike, hence their state variable becomes 1; similarly, spiking neurons become refractory. Of the neurons in the refractory state, those being the ones nearest $\xi _1(t)$, a proportion *p* become quiescent, while the remaining ones remain refractory.
3. At the back of the wave, to the left of $\xi _1(t)$, the interval contains a mixture of neurons in states 0 and $-1$. The former remain at 0 whilst, of the latter, a proportion *p* transition into state 0, with the rest remaining at $-1$ (rules for $x \not \in [\xi _1,\xi _2]$). From this argument, we see that the proportion of refractory neurons in the back of the wave must decrease as $\xi \rightarrow -\pi$.

The resulting mesoscopic variable $J(x,t+1)$ is a spatial translation by $( \xi _2(t) - \xi _1(t) )/3$ of *J*(*x*, *t*). We remark that the approximate transition rules of Fig. 5a are valid also in the case of a bump, albeit the corresponding microstructure does not allow the advection mechanism described above.

**Fig. 6:**
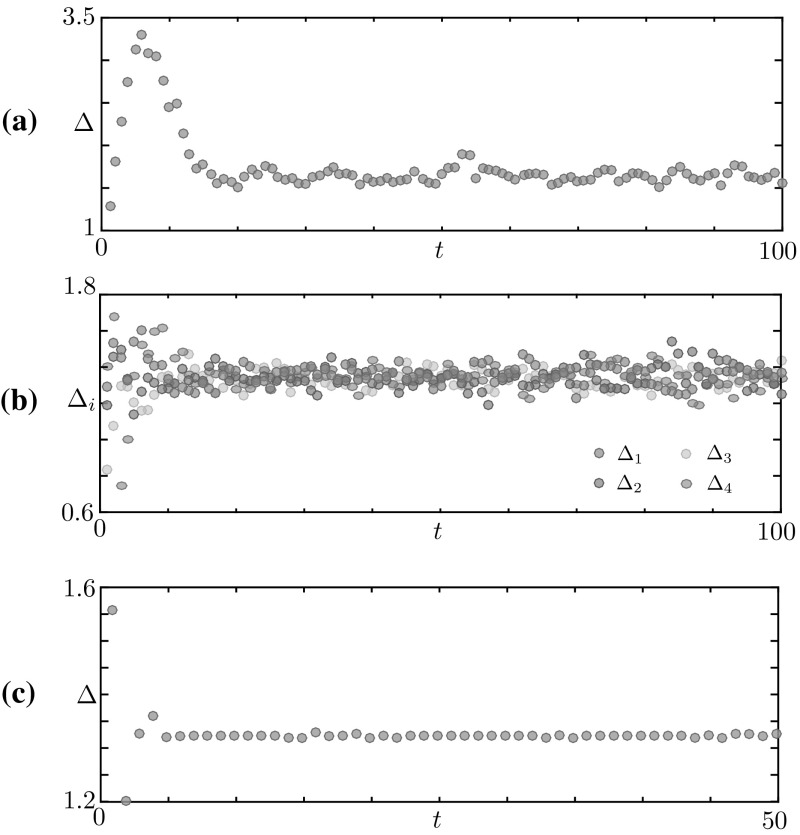
Width of the active regions $\varDelta _i = \xi _{2i} - \xi _{2i-1}$ for the patterns in Figs. 2, 3 and 4. **a** Bump, for which $i=1$. **b** Multiple Bump, $i=1,\ldots ,4$. **c** Travelling wave, $i=1$. In all cases, the patterns reach a coarse equilibrium state after a short transient

### Macroscopic variables

The computations of the previous sections suggest that, beyond the mesoscopic variable, *J*(*x*), coarser macroscopic variables are available to describe the observed patterns. In analogy with what is typically found in neural fields with Heaviside firing rate (Amari 1977; Bressloff 2014; Coombes and Owen 2004), the scalars $\{\xi _i\}$ defining the active region $X_\ge = \cup _i [\xi _{2i-1}, \xi _{2i}]$, where *J* is above *h*, seem plausible macroscopic variables. This is evidenced not only by Figs. 2, 3 and 4, but also from the schematic in Fig. 5b, where the interval $[\xi _1(t),\xi _2(t)]$ is mapped to a new interval $[\xi _1(t+1),\xi _2(t+1)]$ of the same width. To explore this further, we extract the widths $\varDelta _i(t)$ of each sub-interval $[\xi _{2i}(t),\xi _{2i-1}(t)]$ from the data in Figs. 2, 3 and 4, and plot the widths as a function of *t*. In all cases, we observe a brief transient, after which $\varDelta _i(t)$ relaxes towards a coarse equilibrium, though fluctuations seem larger for the bump and multiple bump when compared with those for the wave. In the multiple bump case, we also notice that all intervals have approximately the same asymptotic width (see Fig. 6b).

## Deterministic model

We now introduce a deterministic version of the stochastic model considered in Sect. 2.2, which is suitable for carrying out analytical calculations. We make the following assumptions:

1. *Continuum neural tissue.* We consider the limit of infinitely many neurons and pose the model on $\mathbb {S}$.
2. *Deterministic transitions.* We assume $p=1$, which implies a deterministic transition from refractory states to quiescent ones (see Eq. (5)), and $\beta \rightarrow \infty$, which induces a Heaviside firing rate $f(I) = \Theta (I-h)$ and hence a deterministic transition from quiescent states to spiking ones given sufficiently high input (see Eqs. 4, 6).

In addition to the pullback sets $X_{-1}$, $X_0$, and $X_1$ defined in (2), we will partition the tissue into *active* and *inactive* regions

9 $$\begin{aligned} X_\ge (t) = \{ x \in \mathbb {X}:J(x,t) \ge h \}, \qquad X_<(t) = \mathbb {X}{\setminus } X_\ge (t). \end{aligned}$$

In the deterministic model, the transitions (5)–(7) are then replaced by the following rule

10 $$\begin{aligned} u(x,t+1) = {\left\{ \begin{array}{ll} -1 &{} \text {if }x \in X_1(t), \\ 0 &{} \text {if }x \in X_{-1}(t) \cup \big ( X_0(t) \cap X_<(t) \big ) , \\ 1 &{} \text {if }x \in X_0(t) \cap X_\ge (t) . \end{array}\right. } \end{aligned}$$

We stress that the right-hand side of the equation above depends on *u*(*x*, *t*), since the partitions $\{ X_{-1}, X_0, X_1 \}$ and $\{ X_< , X_\ge \}$ do so (see Remark 1).

As we shall see, it is sometimes useful to refer to the induced mapping of the pullback sets

11 $$\begin{aligned} \begin{aligned} X_{-1}(t+1)&= X_1(t) \\ X_0(t+1)&= X_{-1}(t) \cup \big ( X_0(t) \cap X_<(t) \big ) \\ X_1(t+1)&= X_0(t) \cap X_\ge (t) \end{aligned} \;. \end{aligned}$$

Henceforth, we will use the term *deterministic model* and formally write

12 $$\begin{aligned} u(x,t+1) = \varPhi _\text {d}(u(x,t); \gamma ). \end{aligned}$$

for (10), where the partition $\{ X_k \}_{k \in \mathbb {U}}$ is defined by (2) and the active and inactive sets $X_\ge$, $X_<$ by (9).

**Fig. 7:**
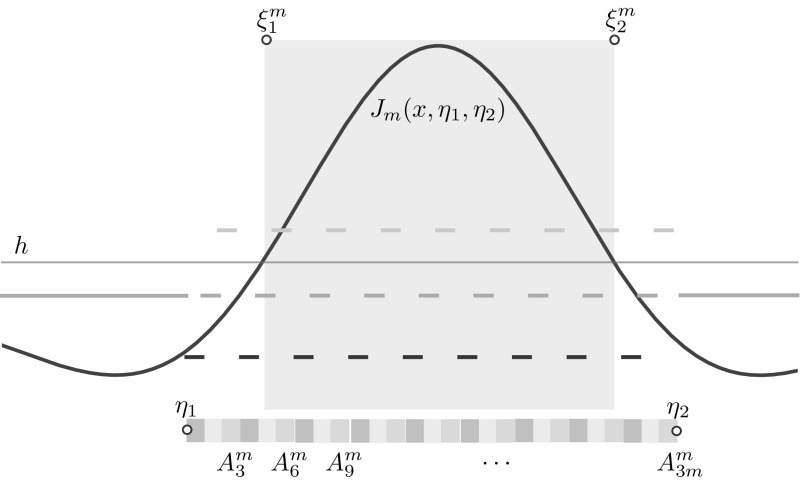
Schematic of the analytical construction of a bump. A microscopic state whose partition comprises $3m+2$ strips is considered. The microscopic state, which is not an equilibrium of the deterministic system, has a characteristic width $\eta _2 - \eta _1$, which differs from the width $\xi ^m_2 -\xi ^m_1$ of the mesoscopic bump $J_m$. If we let $m \rightarrow \infty$ while keeping $\eta _2 - \eta _1$ constant, then $J_m$ tends towards a mesoscopic bump $J_\mathrm {b}$ and $\xi ^m_i \rightarrow \eta _i$ (see Proposition 1)

## Macroscopic bump solution of the deterministic model

We now proceed to construct a bump solution of the deterministic model presented in Sect. 4. In order to do so, we consider a microscopic state with a regular structure, resulting in a partition, $\{X_k^m\}_k$, with $3m+2$ strips (see Fig. 7) and then study the limit $m\rightarrow \infty$.

### Bump construction

Starting from two points $\eta _1, \eta _2 \in \mathbb {S}$, with $\eta _1< \eta _2$, we construct 3*m* intervals as follows

13 $$\begin{aligned} A^m_i = \bigg [ \eta _1 + \frac{i-1}{3m} (\eta _2-\eta _1) , \eta _1 + \frac{i}{3m} (\eta _2 - \eta _1) \bigg ), \qquad i =1,\ldots ,3m, \quad m \in \mathbb {N}. \end{aligned}$$

We then consider states $u_m(x) = \sum _{k \in \mathbb {U}} k {\mathbbm {1}}_{X^m_{k}}(x)$, with partitions given by

14 $$\begin{aligned} X^m_{-1} = \bigcup _{j = 1}^{m} A^m_{3j-2}, \quad X^m_0 = [-L,\eta _1) \cup [\eta _2,L) \bigcup _{j = 1}^{m} A^m_{3j-1}, \quad X^m_1 = \bigcup _{j = 1}^{m} A^m_{3j}, \end{aligned}$$

and activity set $X_\ge = [\xi ^m_1, \xi ^m_2]$. We note that, in addition to the 3*m* strips that form the active region of the bump, we also need two additional strips in the inactive region to form a partition of $\mathbb {S}$. In general, $\{\xi ^m_i\}_i \ne \{\eta _i\}_i$, as illustrated in Fig. 7. Applying (10) or (11), we find $\varPhi _\text {d}(u_m) \ne u_m$, hence $u_m$ are not equilibria of the deterministic model. However, these states help us defining a macroscopic bump as a fixed point of a suitably defined map using the associated mesoscopic synaptic profile

15 $$\begin{aligned} J_m(x,\eta _1,\eta _2) = \kappa \int _{X_1^m(\eta _1,\eta _2)} W(x-y)\, \mathrm {d}y, \end{aligned}$$

where we have highlighted the dependence of $X_1^m$ on $\eta _1,\eta _2$. The proposition below shows that there is a well defined limit, $J_\text {b}$, of the mesoscopic profile as $m \rightarrow \infty$. We also have that $\xi ^m_i \rightarrow \eta _i$ as $m\rightarrow \infty$ and that the threshold crossings of the activity set are roots of a simple nonlinear function.

#### Proposition 1

(Bump construction) Let *W* be the periodic extension of the synaptic kernel (1) and let $h, \kappa \in \mathbb {R}_+$. Further, let $\{ A_i^m \}_{i=1}^{3m}$, $X^m_1$ and $J_m$ be defined as in (13), (14) and (15), respectively, and let $J_\mathrm {b} :\mathbb {S}^3 \rightarrow \mathbb {R}$ be defined as

$$\begin{aligned} J_\mathrm {b}(x,\eta _1,\eta _2) = \frac{\kappa }{3} \int _{\eta _1}^{\eta _2} W(x-y)\, dy. \end{aligned}$$

The following results hold

1. $J_m(x,\eta _1,\eta _2) \rightarrow J_\mathrm {b}(x, \eta _1, \eta _2)$ as $m \rightarrow \infty$ uniformly in the variable *x* for all $\eta _1, \eta _2 \in \mathbb {S}$ with $\eta _1 < \eta _2$,
2. If there exists $\varDelta \in (0,L)$ such that $3h = \kappa \int _0^\varDelta W(y)\, dy$, then
  $$\begin{aligned} J_\mathrm {b}(0,0,\varDelta ) = J_\mathrm {b}(\varDelta ,0,\varDelta ) = h. \end{aligned}$$

#### Proof

We fix $\eta _1<\eta _2$ and consider the 2*L*-periodic continuous mapping $x \mapsto J_\mathrm {b}(x,\eta _1,\eta _2)$, defined on $\mathbb {S}$. We aim to prove that $J_m \rightarrow J_\mathrm {b}$ uniformly in $\mathbb {S}$. We pose

$$\begin{aligned}&I^m_{-1}(x) = \sum _{j=1}^m \int _{A_{3j-2}} W(x-y)\, \mathrm {d}y, \\&I^m_0 (x) = \sum _{j=1}^m \int _{A_{3j-1}} W(x-y)\, \mathrm {d}y, \\&I^m_1 (x) = \sum _{j=1}^m \int _{A_{3j}} W(x-y)\, \mathrm {d}y, \end{aligned}$$

for all $x \in \mathbb {S}$, $m \in \mathbb {N}$. Since the intervals $\{ A_i^m \}_{i=1}^{3m}$ form a partition of $[\eta _1,\eta _2)$ we have

16 $$\begin{aligned} \frac{3}{\kappa } J_\mathrm {b}(x) = I^m_{-1}(x) + I^m_{0}(x) + I^m_{1}(x) \quad \text {for all }x \in \mathbb {S}, m \in \mathbb {N}. \end{aligned}$$

Since *W* is continuous on the compact set $\mathbb {S}$, it is also uniformly continuous on $\mathbb {S}$. Hence, there exists a modulus of continuity $\omega$ of *W*:

$$\begin{aligned} \omega (r) = \sup _{ \begin{array}{c} p,q \in \mathbb {S}\\ |p - q| \le r \end{array} } | W(p) - W(q) |, \qquad \text {with} \lim _{r \rightarrow 0^+} \omega (r) = \omega (0) = 0. \end{aligned}$$

We use $\omega$ to estimate $\vert I_1^m (x) - I_0^m (x) \vert$ as follows:

$$\begin{aligned} \begin{aligned} \vert I_1^m (x) - I_0^m (x) \vert&\le \sum _{j=1}^m \bigg \vert \int _{A_{3j}} W(x-y) \, dy - \int _{A_{3j-1}} W(x-y) \, dy \bigg \vert \\&= \sum _{j=1}^m \bigg \vert \int _{\eta _1 + \frac{3j-1}{3m} (\eta _2-\eta _1)} ^{\eta _1 + \frac{3j}{3m} (\eta _2-\eta _1)} W(x-y) \, dy - \int _{\eta _1 + \frac{3j-2}{3m} (\eta _2-\eta _1)} ^{\eta _1 + \frac{3j-1}{3m} (\eta _2-\eta _1)} W(x-y) \, dy \bigg \vert \\&= \sum _{j=1}^m \bigg \vert \int _{\eta _1 + \frac{3j-1}{3m} (\eta _2-\eta _1)} ^{\eta _1 + \frac{3j}{3m} (\eta _2-\eta _1)} W(x-y) - W\bigg (x-y+ \frac{\eta _2 - \eta _1}{3m} \bigg ) \, dy \bigg \vert \\&\le \sum _{j=1}^m \int _{\eta _1 + \frac{3j-1}{3m} (\eta _2-\eta _1)} ^{\eta _1 + \frac{3j}{3m} (\eta _2-\eta _1)} \bigg \vert W(x-y) - W\bigg (x-y+ \frac{\eta _2 - \eta _1}{3m} \bigg ) \bigg \vert \, dy \\&\le \sum _{j=1}^m \int _{\eta _1 + \frac{3j-1}{3m} (\eta _2-\eta _1)} ^{\eta _1 + \frac{3j}{3m} (\eta _2-\eta _1)} \omega \bigg (\frac{\eta _2 - \eta _1}{3m} \bigg ) \, dy \\&= \omega \bigg (\frac{\eta _2 - \eta _1}{3m} \bigg ) \frac{\eta _2-\eta _1}{3} . \end{aligned} \end{aligned}$$

We have then $\vert I_1^m (x) - I_0^m (x) \vert \rightarrow 0$ as $m \rightarrow \infty$ and since $\omega \big ( (\eta _2-\eta _1)/(3m) \big )$ is independent of *x*, the convergence is uniform. Applying a similar argument, we find $\vert I_{-1}^m (x) - I_0^m (x) \vert \rightarrow 0$ as $m \rightarrow \infty$ and using (16), we conclude $I^m_1, I^m_0, I^m_{-1} \rightarrow J_\mathrm {b}/\kappa$ as $m \rightarrow \infty$. Since $I^m_1 = J_m/\kappa$, then $J_m \rightarrow J_\mathrm {b}$ uniformly for all $x \in \mathbb {S}$ and $\eta _1,\eta _2 \in \mathbb {S}$ with $\eta _1 < \eta _2$, that is, result 1 holds true.

By hypothesis $J_\mathrm {b}(0,0,\varDelta ) = h$ and, using a change of variables under the integral and the fact that *W* is even, it can be shown that $J_\mathrm {b}(\varDelta ,0,\varDelta ) = h$, which proves result 2.

#### Corollary 1

(Bump symmetries) Let $\varDelta$ be defined as in Proposition 1, then $J_\mathrm {b}(x+\delta ,\delta , \delta +\varDelta )$ is a mesoscopic bump for all $\delta \in [L, -\varDelta + L)$. Such bump is symmetric with respect to the axis $x=\delta + \varDelta /2$.

#### Proof

The assertion is obtained using a change of variables in the integral defining $J_\mathrm {b}$ and noting that *W* is even. $\square$

The results above show that, $\xi ^m_i \rightarrow \eta _i$ as $m \rightarrow \infty$, hence we lose the distinction between width of the microscopic pattern, $\eta _2-\eta _1$, and width of the mesoscopic pattern, $\xi ^m_2 - \xi ^m_1$, in that result 2 establishes $J_\mathrm {b}(\eta _i,\eta _1,\eta _2)=h$, for $\eta _1=0$, $\eta _2=\varDelta$. With reference to Fig. 7, the factor 1 / 3 appearing in the expression for $J_\mathrm {b}$ confirms that, in the limit of infinitely many strips, only a third of the intervals $\{A_j^m\}_j$ contribute to the integral. In addition, the formula for $J_\mathrm {b}$ is useful for practical computations as it allows us to determine the width, $\varDelta$, of the bump.

#### Remark 3

(Permuting intervals $A^m_i$) A bump can also be found if the partition $\{X^m_k\}$ of $u_m$ is less regular than the one depicted in Fig. 7. In particular, Proposition 1 can be extended to a more general case of permuted intervals. More precisely, if we consider permutations, $\sigma _j$, of the index sets $\{3j-2,3j-1,3j\}$ for $j=1,\ldots ,m$ and construct partitions

$$\begin{aligned} X^m_{-1} = \bigcup _{j = 1}^{m} A^m_{\sigma _j(3j-2)}, \quad X^m_0 = [-L,0) \cup [\varDelta ,L) \bigcup _{j = 1}^{m} A^m_{\sigma _j(3j-1)}, \quad X^m_1 = \bigcup _{j = 1}^{m} A^m_{\sigma _j(3j)}, \end{aligned}$$

then the resulting $J_m$ converges uniformly to $J_\mathrm {b}$ as $m \rightarrow \infty$. The proof of this result follows closely the one of Proposition 1 and is omitted here for simplicity.

### Bump stability

Once a bump has been constructed, its stability can be studied by employing standard techniques used to analyse neural field models (Bressloff 2012). We consider the map

$$\begin{aligned} \Psi _\mathrm {b} :\mathbb {S}^2 \times \mathbb {S}^2 \rightarrow \mathbb {R}^2, \qquad ( \xi , \eta ) \mapsto \begin{bmatrix} J_\mathrm {b}(\xi _1, \eta _1, \eta _2) - h \\ J_\mathrm {b}(\xi _2, \eta _1, \eta _2) - h \end{bmatrix}. \end{aligned}$$

and study the implicit evolution

17 $$\begin{aligned} \Psi _\mathrm {b}(\xi (t+1),\xi (t)) = 0. \end{aligned}$$

The motivation for studying this evolution comes from Proposition 1, according to which the macroscopic bump $\xi _*=(0,\varDelta )$ is an equilibrium of (17), that is, $\Psi _\mathrm {b}(\xi _*,\xi _*) = 0$. To determine coarse linear stability, we study how small perturbations of $\xi _*$ evolve according to the implicit rule (17). We set $\xi (t) = \xi _* + \varepsilon \widetilde{\xi }(t)$, for $0 < \epsilon \ll 1$ with $\widetilde{\xi }_i = \mathcal {O}(1)$ and expand (17) around $(\xi _*,\xi _*)$, retaining terms up to order $\varepsilon$,

$$\begin{aligned}&\Psi _\mathrm {b}(\xi _*+\varepsilon \widetilde{\xi }(t+1), \xi _*+\varepsilon \widetilde{\xi }(t)) = \Psi _\mathrm {b}(\xi _*, \xi _*) + \varepsilon D_\xi \Psi _\mathrm {b}(\xi _*, \xi _*) \widetilde{\xi }(t+1)\\&\quad + \varepsilon D_\xi \Psi _\mathrm {b}(\xi _*, \xi _*) \widetilde{\xi }(t). \end{aligned}$$

Using the classical ansatz $\tilde{\xi }(t) = \lambda ^t v$, with $\lambda \in \mathbb {C}$ and $v\in \mathbb {S}^2$, we obtain the eigenvalue problem

18 $$\begin{aligned} \lambda \begin{bmatrix} v_1 \\ v_2 \end{bmatrix} = \frac{1}{W(0)-W(\varDelta )} \begin{bmatrix} -W(0)&W(\varDelta ) \\ W(\varDelta )&-W(0) \\ \end{bmatrix} \begin{bmatrix} v_1 \\ v_2 \end{bmatrix}, \end{aligned}$$

with eigenvalues and eigenvectors given by

$$\begin{aligned}&\lambda _1 = \frac{W(\varDelta ) - W(0)}{ W(0) - W(\varDelta )}=-1,&v^1 = (1,1)^T, \\&\lambda _2 = \frac{-W(0) - W(\varDelta )}{ W(0) - W(\varDelta )},&\qquad v^2 = (-1,1)^T. \end{aligned}$$

As expected, we find an eigenvalue with absolute value equal to 1, corresponding to a pure translational eigenvector. The remaining eigenvalue, corresponding to a compression/extension eigenvector, determines the stability of the macroscopic bump. The parameters $A_i$, $B_i$ in Eq. (1) are such that *W* has a global maximum at $x=0$, with $W(0)>0$. Hence, the eigenvalues are finite real numbers and the pattern is stable if $W(\varDelta )<0$. We will present concrete bump computations in Sect. 10.

### Multi-bump solutions

The discussion in the previous section can be extended to the case of solutions featuring multiple bumps. For simplicity, we will discuss here solutions with 2 bumps, but the case of *k* bumps follows straightforwardly. The starting point is a microscopic structure similar to (14), with two disjoint intervals $[\eta _1,\eta _2),[\eta _3, \eta _4) \subset \mathbb {S}$ each subdivided into 3*m* subintervals. We form the vector $\eta =\{\eta _i\}_{i=1}^4$ and have

$$\begin{aligned} \kappa \int _{X^m_1} W(x-y)\, dy = \sum _{j=1}^2 J_{m}(x,\eta _{2j-1},\eta _{2j}) \rightarrow \sum _{j=1}^2 J_\mathrm {b}(x,\eta _{2j-1},\eta _{2j}), \end{aligned}$$

as $m \rightarrow \infty$ uniformly in the variable *x* for all $\eta _1, \ldots , \eta _4 \in \mathbb {S}$ with $\eta _1< \ldots < \eta _4$. In the expression above, $J_m$ and $J_\mathrm {b}$ are the same functions used in Sect. 5.1 for the single bump. In analogy with what was done for the single bump, we consider the mapping defined by

$$\begin{aligned} \Psi :\mathbb {S}^4 \times \mathbb {S}^4 \rightarrow \mathbb {R}^4, \qquad ( \xi , \eta ) \mapsto \bigg \{ -h + \sum _{j=1}^2 J_\mathrm {b}(\xi _i, \eta _{2j-1}, \eta _{2j}) \bigg \}_{i = 1}^4 . \end{aligned}$$

Multi-bump solutions can then be studied as in Sect. 5. We present here the results for a multi-bump for $L=\pi$ with threshold crossings given by

19 $$\begin{aligned} \xi _* = \frac{1}{2} \begin{bmatrix} -\pi - \varDelta \\ -\pi + \varDelta \\ \pi - \varDelta \\ \pi + \varDelta \\ \end{bmatrix}, \end{aligned}$$

where $$\varDelta $$ satisfies

20 $$\begin{aligned} J_\mathrm {b}\bigg (\frac{\pi +\varDelta }{2}, \frac{ -\pi - \varDelta }{2} , \frac{ -\pi + \varDelta }{2} \bigg ) + J_\mathrm {b}\bigg (\frac{\pi +\varDelta }{2}, \frac{ \pi - \varDelta }{2} , \frac{ \pi + \varDelta }{2} \bigg ) = h. \end{aligned}$$

**Fig. 8:**
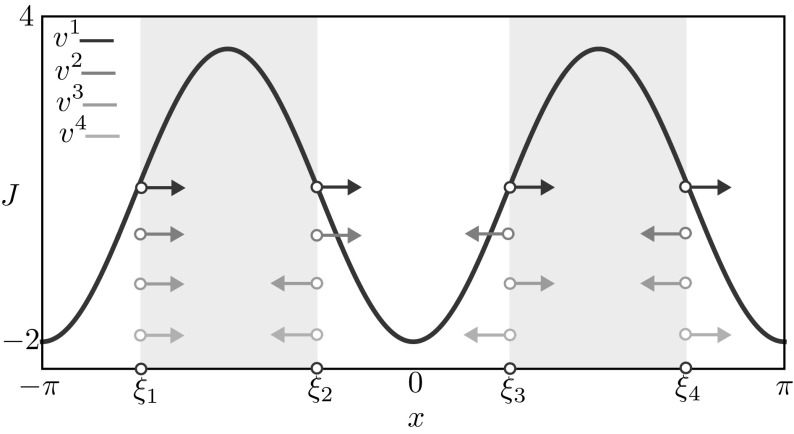
Stable mesoscopic multi-bump obtained for the deterministic model. We also plot the corresponding macroscopic bump $\xi _*$ (Eqs. 19–20) and coarse eigenvectors. Parameters are $\kappa = 30$, $h=0.9$, $p=1$, $\beta \rightarrow \infty$, with other parameters as in Table 1

A quick calculation leads to the eigenvalue problem

21 $$\begin{aligned} \lambda \begin{bmatrix} v_1 \\ v_2 \\ v_3 \\ v_4 \end{bmatrix} = \frac{1}{\alpha } \begin{bmatrix} W(0)&-W(\varDelta )&W(\pi )&-W(\pi - \varDelta ) \\ -W(\varDelta )&W(0)&-W(\pi -\varDelta )&W(\pi ) \\ W(\pi )&-W(\pi -\varDelta )&W(0)&-W(\varDelta ) \\ -W(\pi -\varDelta )&W(\pi )&-W(\varDelta )&W(0) \\ \end{bmatrix} \begin{bmatrix} v_1 \\ v_2 \\ v_3 \\ v_4 \end{bmatrix}, \end{aligned}$$

where $\alpha = -W(0) + W(\varDelta ) - W(\pi ) + W(\pi -\varDelta )$. The real symmetric matrix in Eq. (21) has eigenvalues and eigenvectors given by

$$\begin{aligned}&\lambda _1 = \frac{-W(0) + W(\varDelta )-W(\pi ) + W(\pi -\varDelta )}{ W(0) - W(\varDelta ) + W(\pi ) - W(\pi -\varDelta )}=-1,&v^1 = (1,1,1,1)^T, \\&\lambda _2 = \frac{-W(0) + W(\varDelta ) + W(\pi ) - W(\pi -\varDelta )}{ W(0) - W(\varDelta ) + W(\pi ) - W(\pi -\varDelta )},&v^2 = (1,1,-1,-1)^T, \\&\lambda _3 = \frac{-W(0)-W(\varDelta )-W(\pi )-W(\pi -\varDelta )}{ W(0) - W(\varDelta ) + W(\pi ) - W(\pi -\varDelta )},&v^3 = (1,-1,1,-1)^T, \\&\lambda _4 = \frac{-W(0) - W(\varDelta )+W(\pi ) + W(\pi -\varDelta )}{ W(0) - W(\varDelta ) + W(\pi ) - W(\pi -\varDelta )},&v^4 = (1,-1,-1,1)^T. \end{aligned}$$

As expected, we have one neutral translational mode. If the remaining 3 eigenvalues lie in the unit circle, the multi-bump solution is stable. A depiction of this multi-bump, with corresponding eigenmodes can be found in Fig. 8. We remark that the multi-bump presented here was constructed imposing particular symmetries (the pattern is even; bumps all have the same widths). The system may in principle support more generic bumps, but their construction and stability analysis can be carried out in a similar fashion.

## Travelling waves in the deterministic model

Travelling waves in the deterministic model can also be studied via threshold crossings, and we perform this study in the present section. We seek a measurable function $u_\text {tw} :\mathbb {S}\rightarrow \mathbb {U}$ and a constant $c \in \mathbb {R}$ such that

22 $$\begin{aligned} u(x,t)= u_\text {tw}(x-ct) = \sum _{k \in \mathbb {U}} k {\mathbbm {1}}_{X^\mathrm {tw}_k}(x-ct) \end{aligned}$$

almost everywhere in $\mathbb {S}$ and for all $t \in \mathbb {Z}$. We recall that, in general, a state *u*(*x*, *t*) is completely defined by its partition, $\{ X^\mathrm {tw}_k(t) \}$. Consequently, Eq. (22) expresses that a travelling wave has a fixed profile $u_\text {tw}$, whose partition, $\{ X^\mathrm {tw}_k \}$, does not depend on time. A travelling wave $(u_\text {tw},c)$ satisfies almost everywhere the condition

$$\begin{aligned} u_\text {tw} = \sigma _{-c} \varPhi _\text {d}(u_\text {tw};\gamma ), \end{aligned}$$

where $\varPhi _\text {d}$ is the deterministic evolution operator (12) and the shift operator is defined by $\sigma _{x} :u({\,\cdot \,}) \mapsto u({\,\cdot \,}-x)$. The existence of a travelling wave is now an immediate consequence of the symmetries of *W*, as shown in the following proposition. An important difference with respect to the bump is that analytical expressions can be found for both microscopic and mesoscopic profiles, as opposed to Proposition 1, which concerns only the mesoscopic profile.

### Proposition 2

(Travelling wave) Let $h, \kappa \in \mathbb {R}_+$. If there exists $\varDelta \in (0,L)$ such that $h = \kappa \int _{\varDelta }^{2 \varDelta } W(y)\, dy$, then

$$\begin{aligned} u_\mathrm {tw}(z) = \sum _{k \in \mathbb {U}} k {\mathbbm {1}}_{X^\mathrm {tw}_k}(z), \qquad \text {with partition} \qquad \begin{aligned} X^\mathrm {tw}_{-1}&= [-2\varDelta ,-\varDelta ), \\ X^\mathrm {tw}_{0}&= [-L,-2\varDelta )\cup [0,L), \\ X^\mathrm {tw}_{1}&= [-\varDelta ,0), \end{aligned} \end{aligned}$$

is a travelling wave of the deterministic model (12) with speed $c=\varDelta$, associated mesoscopic profile $J_\mathrm {tw}(z) = \kappa \int _{-\varDelta }^0 W(z-y)\, \mathrm {d}y$ and activity set $X^\mathrm {tw}_\ge = [-2\varDelta ,\varDelta ]$.

### Proof

The assertion can be verified directly. We have

$$\begin{aligned} \frac{h}{\kappa } = \int _\varDelta ^{2\varDelta } w(y) dy = \int _{-\varDelta }^{0} W(\varDelta - y) dy = \int _{-\varDelta }^{0} W(-2\varDelta - y) dy, \end{aligned}$$

hence the activity set for $u_\text {tw}$ is $X^\mathrm {tw}_\ge = [-2\varDelta ,\varDelta ]$ with mesoscopic profile $\kappa \int _{-\varDelta }^0 W(z-y)\, \mathrm {d}y$. Consequently, $\varPhi _\text {d}(u_\text {tw};\gamma )$ has partition

$$\begin{aligned} Y_{-1}&= [-\varDelta ,0), \\ Y_{0}&= [-L,-\varDelta )\cup [\varDelta ,L), \\ Y_{1}&= [0,\varDelta ], \end{aligned}$$

and $u_\text {tw} = \sigma _{-\varDelta }\varPhi _\text {d}(u_\text {tw},\gamma )$ almost everywhere.

Numerical simulations of the deterministic model confirm the existence of the mesoscopic travelling wave $u_\text {tw}$ in a suitable region of parameter space, as will be shown in Sect. 10. The main difference between $u_\text {tw}$ and the stochastic waves observed in Fig. 4 is in the wake of the wave, where the former features quiescent neurons and the latter a mixture of quiescent and refractory neurons.

**Fig. 9:**
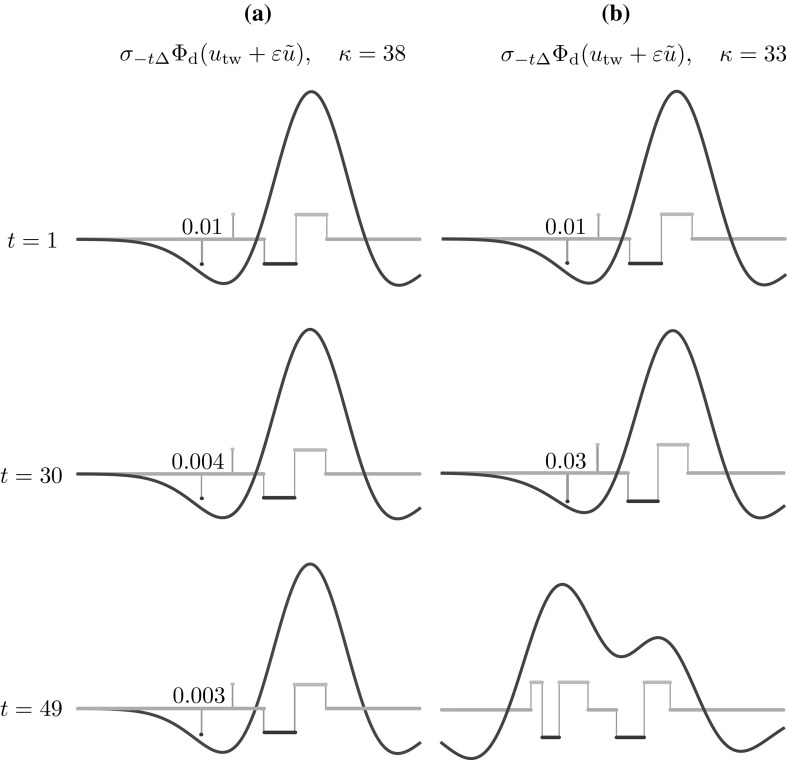
Numerical investigation of the linear stability of the travelling wave of the deterministic system, subject to perturbations in the wake of the wave. We iterate the map $\varPhi _\mathrm {d}$ starting from a perturbed state $u_\mathrm {tw} + \varepsilon \tilde{u}$, where $u_\mathrm {tw}$ is the mesoscopic wave profile of Proposition 2, travelling with speed $\varDelta$, and $\varepsilon \tilde{u}$ is non-zero only in two intervals of width 0.01 in the wake of the wave. We plot $\sigma _{-t\varDelta } \varPhi _\mathrm {d}( u_\mathrm {tw} + \varepsilon \tilde{u})$ and the corresponding macroscopic profile as a function of *t* and we annotate the width of one of the perturbations. **a** For $\kappa =38$, the wave is stable. **b** for sufficiently small $\kappa$, the wave becomes unstable

### Travelling wave stability

As we will show in Sect. 10, waves can be found for sufficiently large values of the gain parameter $\kappa$. However, when this parameter is below a critical value, we observe that waves destabilise at their tail. This type of instability is presented in the numerical experiment of Fig. 9. Here, we iterate the dynamical system

23 $$\begin{aligned} u(z,t+1) = \sigma _{-\varDelta } \varPhi _\text {d}(u(z,t)), \qquad u(z,0) = u_\text {tw}(z) + \varepsilon \widetilde{u}(z), \end{aligned}$$

where $u_\text {tw}$ is the profile of Proposition 2, travelling with speed $\varDelta$, and the perturbation $\varepsilon \widetilde{u}_\text {tw}$ is non-zero only in two intervals of width 0.01. We deem the travelling wave stable if $u(z,t) \rightarrow u_\text {tw}(z)$ as $t \rightarrow \infty$. For $\kappa$ sufficiently large, the perturbations decay, as witnessed by their decreasing width in Fig. 9a. For $\kappa = 33$, the perturbations grow and the wave destabilises.

**Fig. 10:**
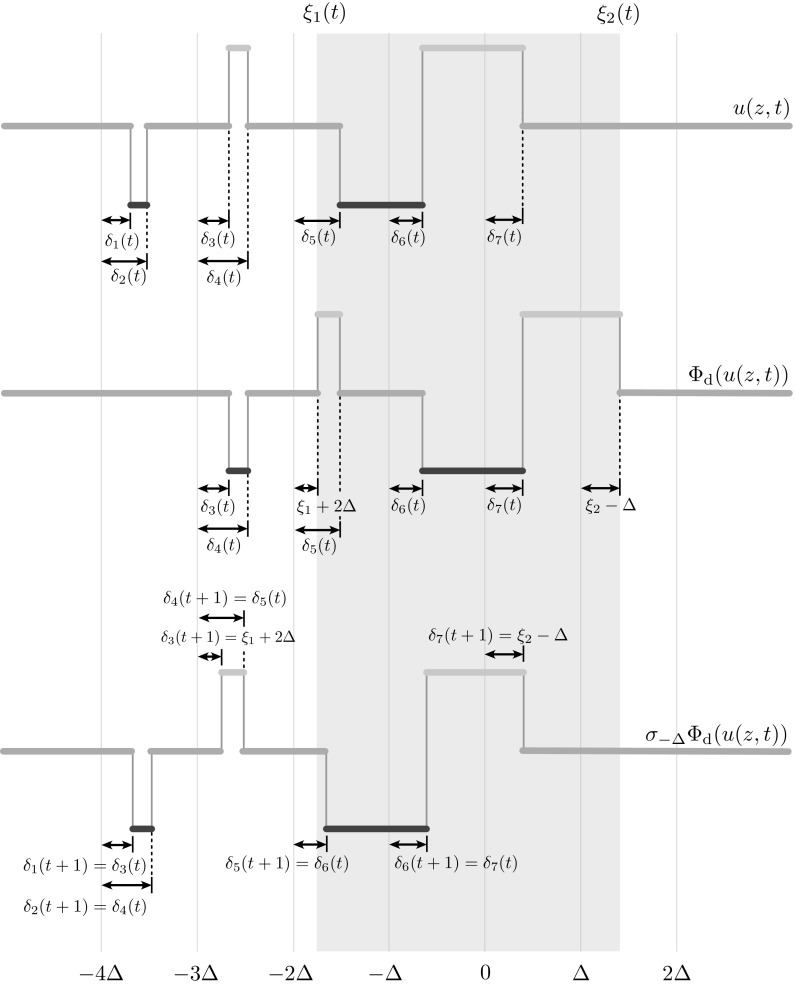
Visualisation of one iteration of the system (23): a perturbed travelling wave (*top*) is first transformed by $\varPhi _\text {d}$ using the rules (11) (*centre*) and then shifted back by an amount $\varDelta$ (*bottom*). This gives rise to an implicit evolution equation $\Psi \big (\delta (t+1),\delta (t)\big )=0$ for the threshold crossing points of the perturbed wave, as detailed in the text

To analyse the behaviour of Fig. 9, we shall derive the evolution equation for a relevant class of perturbations to $u_\text {tw}$. This class may be regarded as a generalisation of the perturbation applied in this figure and is sufficient to capture the instabilities observed in numerical simulations. We seek solutions to (23) with initial condition $u(z,t) = \sum _{k} k {\mathbbm {1}}_{X_k(t)}(z)$ with time-dependent partitions

$$\begin{aligned}&X_{-1}(t) = \Big [-4\varDelta + \delta _1(t),-4\varDelta + \delta _2(t) \Big ) \cup \Big [-2\varDelta + \delta _5(t),- \varDelta + \delta _6(t) \Big ),\\&\begin{aligned} X_0(t)&= \Big [-L,-4\varDelta + \delta _1(t) \Big ) \cup \Big [-4\varDelta + \delta _2(t),-3\varDelta + \delta _3(t) \Big )\\&\phantom {= \Big (-L,-4\varDelta + \delta _1(t) \Big ) } \, \cup \Big [-3\varDelta + \delta _4(t),-2\varDelta + \delta _5(t) \Big ) \cup \Big [\delta _7(t), L \Big ), \end{aligned} \\&X_1(t) = \Big [-3\varDelta + \delta _3(t),-3\varDelta + \delta _4(t) \Big ) \cup \Big [ -\varDelta + \delta _6(t),\delta _7(t) \Big ), \end{aligned}$$

and activity set $X_\ge (t) = [\xi _1(t),\xi _2(t)]$. In passing, we note that for $\delta _i =0$, the partition above coincides with $\{X^\mathrm {tw}_k\}$ in Proposition 2, hence this partition can be used as perturbation of $u_\text {tw}$. Inserting the ansatz for $u(\xi ,t)$ into (23), we obtain a nonlinear implicit evolution equation, $\Psi \big (\delta (t+1),\delta (t)\big )=0$, for the vector $\delta (t)$ as follows (see Fig. 10)

$$\begin{aligned}&\delta _1(t+1) = \delta _3(t),\\&\delta _2(t+1) = \delta _4(t), \\&\int _{-3\varDelta + \delta _3(t)}^{-3\varDelta + \delta _4(t)} w(-2\varDelta + \delta _3(t+1) - y) \, dy + \int _{-\varDelta + \delta _6(t)}^{\delta _7(t)} w(-2\varDelta + \delta _3(t+1) - y) \, dy = h/\kappa ,\\&\delta _4(t+1) = \delta _5(t), \\&\delta _5(t+1) = \delta _6(t), \\&\delta _6(t+1) = \delta _7(t), \\&\int _{-3\varDelta + \delta _3(t)}^{-3\varDelta + \delta _4(t)} w(\varDelta + \delta _7(t+1) - y) \, dy + \int _{-\varDelta + \delta _6(t)}^{\delta _7(t)} w(\varDelta + \delta _7(t+1) - y) \, dy = h/\kappa . \end{aligned}$$

We note that the map above is valid under the assumption $\delta _3(t) < \delta _4(t)$, which preserve the number of intervals of the original partition. As in Kilpatrick and Bressloff (2010), we note that this prevents us from looking at oscillatory evolution of $\delta (t)$. We set $\delta _i(t) = \varepsilon \lambda ^t v_i$, retain terms up to first order and obtain an eigenvalue problem for the matrix

$$\begin{aligned} \frac{1}{\alpha } \begin{bmatrix} 0&0&\alpha&0&0&0&0 \\ 0&0&0&\alpha&0&0&0 \\ 0&0&-w(\varDelta )&w(\varDelta )&0&-w(\varDelta )&w(2\varDelta ) \\ 0&0&0&0&\alpha&0&0 \\ 0&0&0&0&0&\alpha&0 \\ 0&0&0&0&0&0&\alpha \\ 0&0&w(4\varDelta )&-w(4\varDelta )&0&w(2\varDelta )&-w(\varDelta ) \\ \end{bmatrix}, \end{aligned}$$

where $\alpha = w(2\varDelta ) - w(\varDelta )$. Once again, we have an eigenvalue on the unit circle, corresponding to a neutrally stable translation mode. If all other eigenvalues are within the unit circle, then the wave is linearly stable. Concrete calculations will be presented in Sect. 10.

## Approximate probability mass functions for the Markov chain model

We have thus far analysed coherent states of a deterministic limit of the Markov chain model, and we now move to the more challenging stochastic setting. More precisely, we return to the original model (8) and find *approximate* mass functions for the coherent structures presented in Sect. 3 (see Figs. 2, 3, 4). These approximations will be used in the lifting procedure of the equation-free framework.

The stochastic model is a Markov chain whose $3^N$-by-$3^N$ transition kernel has entries specified by (1). It is useful to examine the evolution of the probability mass function for the state of a neuron at position $x_i$ in the network, $\mu _k(x_i,t) = {{\mathrm{Pr}}}\big ( u(x_i,t) = k \big )$, $k \in \mathbb {U}$, which evolves according to

24 $$\begin{aligned} \begin{bmatrix} \mu _{-1} (x_i,t+1) \\ \mu _0 (x_i,t+1) \\ \mu _1 (x_i,t+1) \\ \end{bmatrix}= \begin{bmatrix} 1-p&0&1 \\ p&1-f(J(u))(x_i,t)&0 \\ 0&f(J(u))(x_i,t)&0 \end{bmatrix} \begin{bmatrix} \mu _{-1} (x_i,t) \\ \mu _0 (x_i,t) \\ \mu _1 (x_i,t) \\ \end{bmatrix}, \end{aligned}$$

or in compact notation $\mu (x_i,t+1) = \Pi (x_i,t) \mu (x_i,t)$. We recall that *f* is the sigmoidal firing rate and that *J* is a deterministic function of the random vector, $u(x,t) \in \mathbb {U}^N$, via the pullback set $X^u_1(t)$:

$$\begin{aligned} J(u)(x,t) = \kappa \int _{\mathbb {X}} W(x - y) {\mathbbm {1}}_{X^u_1(t)}(y) \, \mathrm {d}y. \end{aligned}$$

As a consequence, the evolution equation for $\mu (x_i,t)$ is non-local, in that $J(x_i,t)$ depends on the microscopic state of the whole network.

We now introduce an approximate evolution equation, obtained by posing the problem on a continuum tissue $\mathbb {S}$ and by substituting *J*(*x*, *t*) by its expected value

25 $$\begin{aligned} \mu (x,t+1) = \widetilde{\Pi }(x,t) \mu (x,t), \end{aligned}$$

where $$\mu :\mathbb {S}\times \mathbb {Z}\rightarrow [0,1]^3$$,

26 $$\begin{aligned} \widetilde{\Pi }(x,t) = \begin{bmatrix} 1-p&0&1 \\ p&1-f\big (\mathbb {E}[J]\big )(x,t)&0 \\ 0&f\big (\mathbb {E}[J]\big )(x,t)&0 \end{bmatrix}, \end{aligned}$$

and

27 $$\begin{aligned} \mathbb {E}[J](x,t) = \kappa \int _\mathbb {S}w(x-y) \mu _1(y,t)\, \mathrm {d}y. \end{aligned}$$

In passing, we note that the evolution Eq. (25) is deterministic. We are interested in two types of solutions to (25):

1. A time-independent bump solution, that is a mapping $\mu _\text {b}$ such that $\mu (x,t)=\mu _\text {b}(x)$ for all $x \in \mathbb {S}$ and $t\in \mathbb {Z}$.
2. A travelling wave solution, that is, a mapping $\mu _\text {tw}$ and a real number *c* such that $\mu (x,t)=\mu _\text {tw}(x - ct)$ for all $x \in \mathbb {S}$ and $t\in \mathbb {Z}$.

### Approximate probability mass function for bumps

We observe that, posing $\mu (y,t)=\mu _{\text {b}}(y)$ in (25), we have

$$\begin{aligned} \mathbb {E}[J](x) = \kappa \int _\mathbb {S}w(x-y) (\mu _{\text {b}})_1(y)\, \mathrm {d}y . \end{aligned}$$

Motivated by the simulations in Sect. 3 and by Proposition 1, we seek a solution to (25) in the limit $\beta \rightarrow \infty$, with $\mathbb {E}[J](x) \ge h$ for $x \in [0,\varDelta ]$, and $(\mu _\text {b})_1(x) \ne 0$ for $x \in [0,\varDelta ]$, where $\varDelta$ is unknown. We obtain

$$\begin{aligned} \mu _\text {b}(x) = \widetilde{\Pi }_\text {b}(x) \mu _\text {b}(x), \end{aligned}$$

where

$$\begin{aligned} \begin{aligned} \widetilde{\Pi }_\text {b}(x)&= \begin{bmatrix} 1-p&0&1 \\ p&1&0 \\ 0&0&0 \end{bmatrix} {\mathbbm {1}}_{\mathbb {S}\setminus [0,\varDelta ]}(x) + \begin{bmatrix} 1-p&0&1 \\ p&0&0 \\ 0&1&0 \end{bmatrix} {\mathbbm {1}}_{[0,\varDelta ]}(x)\\&= Q_< {\mathbbm {1}}_{\mathbb {S}\setminus [0,\varDelta ]}(x) + Q_\ge {\mathbbm {1}}_{[0,\varDelta ]}(x),\\ \end{aligned} \end{aligned}$$

We conclude that, for each $x \in [0,\varDelta ]$ (respectively $x \in \mathbb {S}{\setminus } [0,\varDelta ]$), $\mu _\text {b}(x)$ is the right $\Vert {\,\cdot \,}\Vert _1$-unit eigenvector corresponding to the eigenvalue 1 of the stochastic matrix $Q_\ge$ (respectively $Q_<$). We find

28 $$\begin{aligned} \mu _\text {b}(x) = \begin{bmatrix} 0 \\ 1 \\ 0 \end{bmatrix} {\mathbbm {1}}_{\mathbb {S}\setminus [0,\varDelta ]}(x) + \frac{p}{1+ 2p} \begin{bmatrix} 1/p \\ 1 \\ 1 \end{bmatrix} {\mathbbm {1}}_{[0,\varDelta ]}(x) \end{aligned}$$

and, by imposing the threshold condition $\mathbb {E}[J](\varDelta ) = h$, we obtain a compatibility condition for $\varDelta$,

29 $$\begin{aligned} h = \frac{\kappa p}{1+2p} \int _0^\varDelta w(\varDelta -y)\, \mathrm {d}y. \end{aligned}$$

We note that if $p=1$ we have $\mathbb {E}[J](x) = J_\text {b}(x,0,\varDelta )$ where $J_\text {b}$ is the profile for the mesoscopic bump found in Proposition 1, as expected.

**Fig. 11:**
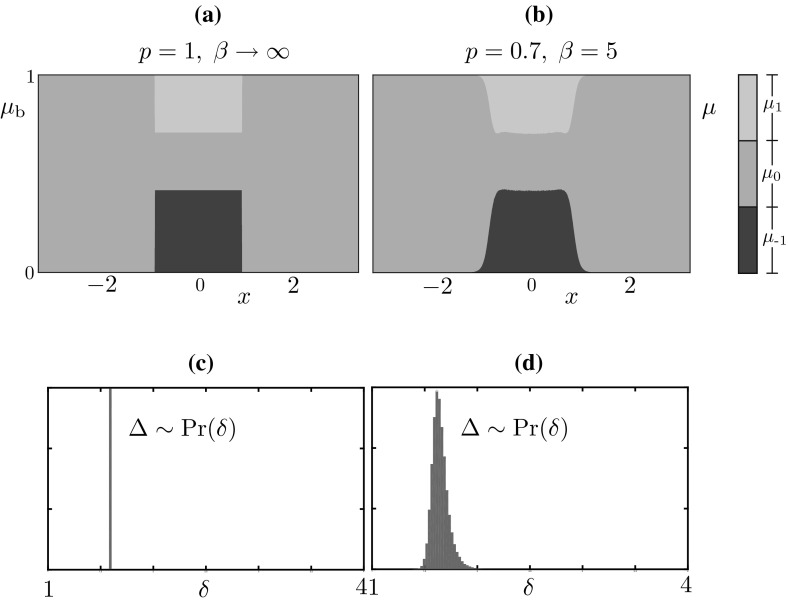
Comparison between the probability mass function $\mu _\mathrm {b}$, as computed by (28)–(29), and the observed distribution $\mu$ of the stochastic model. **a** We compute the vector $(\mu _\mathrm {b})_k$, $k\in \mathbb {U}$ in each strip using (30) and visualise the distribution using vertically juxtaposed color bars, with height proportional to the values $(\mu _\mathrm {b})_k$, as shown in the legend. **b** A long simulation of the stochastic model supporting a stochastic bump *u*(*x*, *t*) for $t \in [0,T]$, where $T=10^5$. At each time $t>10$ (allowing for initial transients to decay), we compute $\xi _1(t)$, $\xi _2(t)$, $\varDelta (t)$ and then produce histograms for the random profile $u(x-\xi _1(t) - \varDelta (t)/2,t)$. **c** In the deterministic limit, the value of $\varDelta$ is determined by (29), hence we have a Dirac distribution. **d** the distribution of $\varDelta$ obtained in the Markov chain model. Parameters are as in Table 1

In Fig. 11a, we plot $\mu _\mathrm {b}(x)$ as predicted by (28)–(29), for $p = 0.7,\,\kappa = 30,\,h=0.9$. At each *x*, we visualise $(\mu _\text {b})_k$ for each $k \in \mathbb {U}$ using vertically juxtaposed color bars, with height proportional to the values $(\mu _\text {b})_k$, as shown in the legend. For a qualitative comparison with direct simulations, we refer the reader to the microscopic profile *u*(*x*, 50) shown in the right panel of Fig. 2a: the comparison suggests that each $u(x_i,50)$ is distributed according to $\mu _\text {b}(x_i)$.

We also compared quantitatively the approximate distribution $\mu _\text {b}$ with the distribution, $\mu (x,t)$, obtained via Monte Carlo samples of the full system (24). The distributions are obtained from a long-time simulation of the stochastic model supporting a microscopic bump *u*(*x*, *t*) for $t \in [0,T]$, with $T=10^5$. At each discrete time *t*, we compute the mesoscopic profile, *J*(*u*)(*x*, *t*), the corresponding threshold crossings and width: $\xi _1(t)$, $\xi _2(t)$, $\varDelta (t)$ and then produce histograms for the random profile $u(x-\xi _1(t) - \varDelta (t)/2,t)$. The instantaneous shift applied to the profile is necessary to pin the wandering bump.

We note a discrepancy between the analytically computed histograms, in which we observe a sharp transition between the region $x\in [0,\varDelta ]$ and $x\in \mathbb {S}{\setminus } [0,\varDelta ]$, and the numerically computed ones, in which this transition is smoother. This discrepancy arises because $\varDelta (t)$ oscillates around an average value $\varDelta$ predicted by (29); the approximate evolution Eq. (25) does not account for these oscillations. This is visible in the histograms of Fig. 11c, d, as well as in the direct numerical simulation Fig. 6a.

### Approximate probability mass function for travelling waves

We now follow a similar strategy to approximate the probability mass function for travelling waves. We pose $\mu (x,t) = \mu _\text {tw}(x-ct)$ in the expression for $\mathbb {E}[J]$, to obtain

$$\begin{aligned} \kappa \int _\mathbb {S}w(x-y) (\mu _{\text {tw}})_1(y-ct)\, \mathrm {d}y = \kappa \int _\mathbb {S}w(x-ct-y) (\mu _{\text {tw}})_1(y)\, \mathrm {d}y = \mathbb {E}[J](x-ct). \end{aligned}$$

Proposition 2 provides us with a deterministic travelling wave with speed $c=\varDelta$. The parameter $\varDelta$ is also connected to the mesoscopic wave profile, which has threshold crossings $\xi _1= -2\varDelta$ and $\xi _2=\varDelta$. Hence, we seek for a solution to (25) in the limit $\beta \rightarrow \infty$, with $\mathbb {E}[J](z) \ge h$ for $x \in [-2c,c]$, and $(\mu _\text {tw})_1(z) \ne 0$ for $z \in [-2c,c]$, where *c* is unknown. For simplicity, we pose the problem on a large domain whose size is commensurate with *c*, that is $\mathbb {S}=cT/\mathbb {R}$, where *T* is an even integer much greater than 1.

We obtain

$$\begin{aligned} \sigma _{ct} \mu _\text {tw}(z) = \widetilde{\Pi }_\text {tw}(z - c(t-1)) \widetilde{\Pi }_\text {tw}(z - c(t-2)) \cdots \widetilde{\Pi }_\text {tw}(z) \mu _\text {tw}(z), \end{aligned}$$

where

$$\begin{aligned} \widetilde{\Pi }_\text {tw}(z) = Q_< {\mathbbm {1}}_{\mathbb {S}\setminus [-c,c]}(z) + Q_\ge {\mathbbm {1}}_{\mathbb {S}[-2c,c]}(z). \end{aligned}$$

To make further analytical progress, it is useful to partition the domain $\mathbb {S}=cT/\mathbb {R}$ in strips of width *c*,

$$\begin{aligned} \mathbb {S}= \bigcup _{j=T/2}^{T/2} \, \big [ j c, (j+1) c \big ) = \bigcup _{j=T/2}^{T/2-1} I_j(c), \end{aligned}$$

and impose that the wave returns back to its original position after *T* iterations, $\sigma _{cT} \mu _\text {tw}(z)= \mu _\text {tw}(z)$, while satisfying the compatibility condition $h = \mathbb {E}[J](c)$. This leads to the system

30 $$\begin{aligned} \begin{aligned}&\mu _\text {tw}(z) = R(z,c) \mu _\text {tw}(z) = \Bigg [ \sum _{j=-T/2}^{T/2-1} R_j {\mathbbm {1}}_{I_j(c)}(z) \Bigg ] \mu _\text {tw}(z), \\&\kappa \int _{-2c}^c W(c-y) (\mu _\text {tw})_1(y)\, \mathrm {d}y = h. \end{aligned} \end{aligned}$$

With reference to system (30) we note that:

1. *R*(*z*, *c*) is constant within each strip $I_j$, hence the probability mass function, $\mu _\text {tw}(z)$, is also constant in each strip, that is, $\mu _\text {tw}(z) = \sum _i \rho _i {\mathbbm {1}}_{I_i(c)}(z)$ for some unknown vector $(\rho _{-T/2},\ldots ,\rho _{T/2}) \in \mathbb {S}^{3T}$.
2. Each $R_j$ is a product of *T* 3-by-3 stochastic matrices, each equal to $Q_<$ or $Q_\ge$. Furthermore, the matrices $\{ R_j \}$ are computable. For instance, for the strip $I_{-1}$ we have
  $$\begin{aligned} R_{-1}= \begin{bmatrix} (1-p)^{T} + p(1-p)^{T-2}&(1-p)^{T-2}&(1-p)^{T-1} \\ p(1-p)^{T-1} + p^2(1-p)^{T-3}&p(1-p)^{T-3}&p(1-p)^{T-2} \\ 1-(1-p)^{T-1}-p(1-p)^{T-3}&1-(1-p)^{T-3}&1-(1-p)^{T-2} \\ \end{bmatrix}. \end{aligned}$$

Consequently, $\mu _\text {tw}(z)$ can be determined by solving the following problem in the unknown $(\rho _{-T/2},\ldots ,\rho _{T/2},c) \in \mathbb {S}^{3T} \times \mathbb {R}$:

31 $$\begin{aligned} \begin{aligned} \rho _i - R_i \rho _i&= 0, \qquad&i = -T/2,\ldots ,T/2-1, \\ - h + \kappa (\rho _{-1})_1 \displaystyle {\int _{-c}^0} W(c-y) \, \mathrm {d}y&= 0.&\end{aligned} \end{aligned}$$

**Fig. 12:**
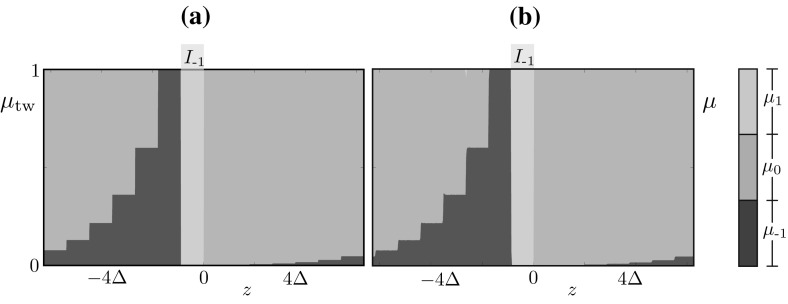
Similarly to Fig. 11, we compare the approximated probability mass function $\mu _\text {tw}$, and the observed distribution $\mu$ of the stochastic model. **a** The probability mass function is approximated using the numerical scheme outlined in the main text for the solution of (31); the strip $I_{-1}$ is indicated for reference. **b** A set of $9\times 10^5$ realisations of the stochastic model for a travelling wave are run for $t \in [0,T]$, where $T=1000$. For each realisation *s*, we calculate the final threshold crossings $\xi ^s_1(T)$, $\xi ^s_2(T)$, and then compute histograms of $u^s(x-\xi ^s_2(T),T)$. We stress that the strips in (**a**) are induced by our numerical procedure, while the ones in (**b**) emerge from the data. The agreement is excellent and is preserved across a vast region of parameter space (not shown). Parameters are as in Table 1

Before presenting a quantitative comparison between the numerically determined distribution, $\mu _\text {tw}(z)$, and that obtained via direct time simulations, we make a few efficiency considerations. In the following sections, it will become apparent that sampling the distribution $\mu _\text {tw}(z)$ for various values of control parameters, such as *h* or $\kappa$, is a recurrent task, at the core of the coarse bifurcation analysis: each linear and nonlinear function evaluation within the continuation algorithm requires sampling $\mu _\text {tw}(z)$, and hence solving the large nonlinear problem (31).

With little effort, however, we can obtain an accurate *approximation* to $\mu _\text {tw}$, with considerable computational savings. The inspiration comes once again from the analytical wave of Proposition 2. We notice that only the last equation of system (31) is nonlinear; the last equation is also the only one which couples $\{\rho _j\}$ with *c*. When $p=1$ the wave speed is known as $\beta \rightarrow \infty$, $N\rightarrow \infty$ and $p=1$ corresponds to the deterministic limit, hence $\mathbb {E}[J](z) = J_\text {tw}(z)$, which implies $c = \varDelta$ and $(\rho _{-1})_1 = 1$. The stochastic waves observed in direct simulations for $p\ne 1$, however, display $c \approx (\xi _2 - \xi _1)/3 = \varDelta$ and $\mu \approx 1$ in the strip where *J* achieves a local maximum (see, for instance Fig. 4, for which $p = 0.4$).

The considerations above lead us to the following scheme to approximate $\mu _\text {tw}$: (i) set $c = \varDelta$ and remove the last equation in (31); (ii) solve *T* decoupled 3-by-3 eigenvalue problems to find $\rho _i$. Furthermore, if *p* remains fixed in the coarse bifurcation analysis, $\rho _i$ can be pre-computed and step (ii) can be skipped.

In Fig. 12a, we report the approximate $\mu _\text {tw}$ found with the numerical procedure described above. An inspection of the microscopic profile *u*(*x*, 45) in the right panel of Fig. 4a shows that this profile is compatible with $\mu _\text {tw}$. We also compared quantitatively the approximate distribution with the distribution, $\mu (x,t)$, obtained with Monte Carlo samples of the full system (24). The distributions are obtained from *M* samples $\{u^s(x,t)\}^M_{s=1}$ of the stochastic model for a travelling wave for $t \in [0,T]$. For each sample *s*, we compute the thresholds, $\xi ^s_1(T)$, $\xi ^s_2(T)$, of the corresponding $J(u^s)(x,T)$ and then produce histograms for $u^s(x-\xi ^s_2(T),T)$. This shifting, whose results are reported in Fig. 12b, does not enforce any constant value for the velocity, hence it allows us to test the numerical approximation $\mu _\text {tw}$. The agreement between the two distributions is excellent: we stress that, while the strips in Fig. 12a are enforced by our approximation, the ones in Fig. 12b emerge from the data. We note a slight discrepancy, in that $\mu _\text {tw}(-3\varDelta ) \approx 0$, while the other distribution shows a small nonzero probability attributed to the firing state at $\xi =-3\varDelta$. Despite this minor disagreement, the differences between the approximated and observed distributions remain small across all parameter regimes of note and the approximations even retain their accuracy as $\beta$ is decreased (not shown).

## Coarse time-stepper

As mentioned in the introduction, equation-free methods allow us to compute macroscopic states in cases in which a macroscopic evolution equation is not available in closed form (Kevrekidis et al. 2003; Kevrekidis and Samaey 2009). To understand the general idea behind the equation-free framework, we initially discuss an example taken from one of the previous sections, where an evolution equation *does* exist in closed form.

In Sect. 5, we described bumps in a deterministic limit of the Markov chain model. In this description, we singled out a *microscopic* state (the function $u_m(x)$ with partition (14)) and a corresponding *mesoscopic* state (the function $J_m(x)$), both sketched in Fig. 7. Proposition 1 shows that there exists a well defined mesoscopic limit profile, $J_\text {b}$, which is determined (up to translations in *x*) by its threshold crossings $\xi _1=0$, $\xi _2=\varDelta$. This suggests a characterisation of the bump in terms of the *macroscopic* vector $(\xi _1,\xi _2)$ or, once translation invariance is factored out, in terms of the *macroscopic* bump width, $\varDelta$. Even though the microscopic state $u_m$ is not an equilibrium of the deterministic system, the macroscopic state $(0,\varDelta )$ is a fixed point of the evolution Eq. (17), whose evolution operator $\Psi$ is known in closed form, owing to Proposition 1. It is then possible to compute $\varDelta$ as a root of an explicitly available nonlinear equation.

We now aim to use equation-free methods to compute macroscopic equilibria in cases where we do not have an explicit evolution equation, but only a numerical procedure to approximate $\Psi$. As mentioned in the introduction, the evolution equation is approximated using a coarse time-stepper, which maps the macroscopic state at time $t_0$ to the macroscopic state at time $t_1$ using three stages: lifting, evolution, restriction. The specification of these stages (the lifting in particular) typically requires some closure assumptions, which are enforced numerically. In our case, we use the analysis of the previous sections for this purpose. In the following section, we discuss the coarse time-stepper for bumps and travelling waves. The multi-bump case is a straightforward extension of the single bump case.

### Coarse time-stepper for bumps

The macroscopic variables for the bump are the threshold crossings $\{\xi _i\}$ of the mesoscopic profile *J*. The lifting operator for the bump takes as arguments $\{ \xi _i \}$ and returns a set of microscopic profiles compatible with these threshold crossings:

$$\begin{aligned} \mathcal {L}_\text {b} :\mathbb {S}^2 \rightarrow \mathbb {U}^{N \times M}, \qquad (\xi _1,\xi _2)^\mathrm {T} \mapsto \{ u^s(x) \}_s. \end{aligned}$$

**Fig. 13:**
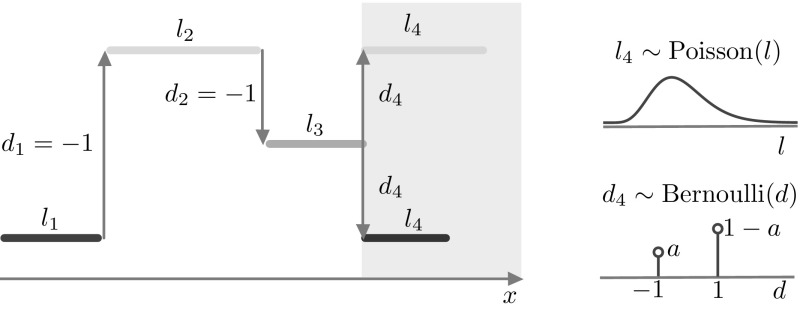
Schematic representation of the lift operator for a bump solution. This figure displays a representation of how the states for neurons located within the activity set, $[\xi _1,\xi _2]$, are lifted. For illustrative purposes, we assume here that we are midway through the lifting operation, where 3 steps of the *while* loop listed in Algorithm 1 have been completed and a fourth one is being executed (shaded area). The width $l_4$ of the next strip is drawn from a Poisson distribution. The random variable $d\in \lbrace -1, 1\rbrace$ indicates the direction through which we cycle through the states $\lbrace -1, 0, 1 \rbrace$ during the lifting. The number $d_4$ is drawn from a Bernoulli distribution whose average *a* gives the probablity of changing direction. For full details of the lifting operator, please refer to Algorithm 1

If $\beta \rightarrow \infty$, $u^s(x)$ are samples of the analytical probability mass function $\mu _\text {b}(x + \varDelta /2)$, where $\mu _\text {b}$ is given by (28) with $\varDelta =\xi _2-\xi _1$. In this limit, a solution branch may also be traced by plotting (29).

If $\beta$ is finite, we either extract samples from the *approximate* probability mass function $\mu _\text {b}$ used above, or we extract samples $u^s(x)$ satisfying the following properties (see Proposition 1 and Remark 3):

1. $u^s(x)$ is symmetric with respect to the axis $x = (\widetilde{\xi }_1 + \widetilde{\xi }_2)/2$, where $\tilde{\xi }_i = {{\mathrm{round}}}(\xi _i)$ and ${{\mathrm{round}}}:\mathbb {S}\rightarrow \mathbb {S}_N$.
2. $$u^s(x) = 0$$ for all $$x \in [-L,\widetilde{\xi }_1) \cup (\widetilde{\xi }_2,L)$$.
3. The pullback sets, $X_1$ and $X_{-1}$, are contained within $[\widetilde{\xi }_1, \widetilde{\xi }_2]$ and are unions of a random number of intervals whose widths are also random. A schematic of the lifting operator for bumps is shown in Fig. 13.

A more precise description of the latter sampling is given in Algorithm 1. As mentioned in the introduction, lifting operators are not unique and we have given above two possible examples of lifting. In our computations, we favour the second sampling method. The mesoscopic profiles, *J*, generated using this approach are well-matched to $\mathbb {E}[J]$ produced by the analytically derived probability mass functions (28). Numerical experiments demonstrate that this method is better than the first possible lifting choice at continuing unstable branches. This is most likely due to the fact that the latter method slightly overestimates the probability of neurons within the bump to be in the spiking state, and underestimates that of them being in the refractory state and this helps mitigate the problems encountered when finding unstable states caused by the combination of the finite size of the network and non-smooth characteristics of the model (when $\beta$ is high).
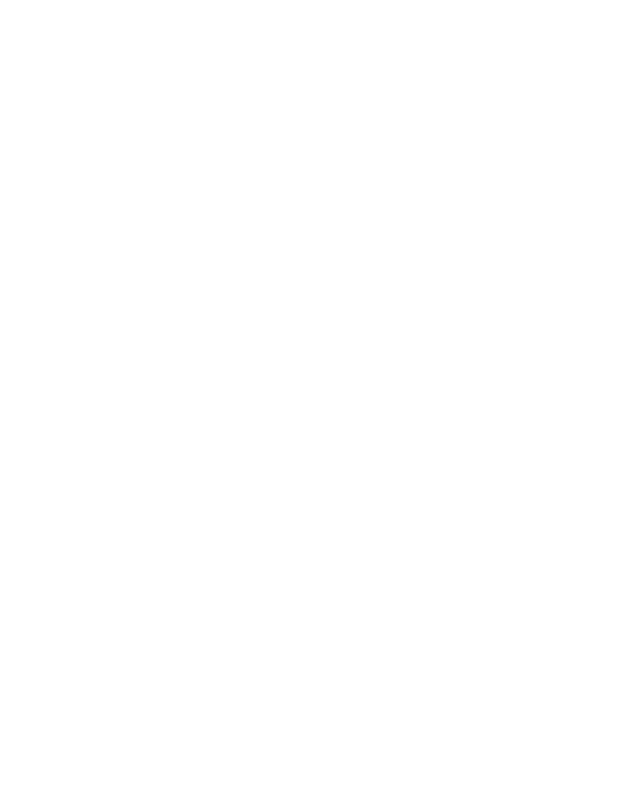


The evolution operator is given by

$$\begin{aligned} \mathcal {E}_T :\mathbb {U}^{N \times M} \rightarrow \mathbb {U}^{N \times M}, \qquad \{ u^j(x) \}_j \mapsto \{ \varphi _T( u^j(x)) \}_j, \end{aligned}$$

where $\varphi _T$ denotes *T* compositions of the microscopic evolution operator (8) and the dependence on the control parameter, $\gamma$, is omitted for simplicity.

For the restriction operator, we compute the average activity set of the profiles. More specifically, we set

$$\begin{aligned} \mathcal {R} :\mathbb {U}^{N \times M} \rightarrow \mathbb {S}^2, \qquad \{ u^j(x) \}_j \mapsto ( \xi _1, \xi _2 )^\mathrm {T}, \end{aligned}$$

where

$$\begin{aligned} \xi _i = \frac{1}{M} \sum _{s=1}^M \xi _i^s, \quad i = 1,2, \end{aligned}$$

and $\xi ^s_i$ are defined using a piecewise first-order interpolant $\mathcal {P}^3_N J$ of *J* with nodes $\{x_i\}_{i=0}^N$,

$$\begin{aligned}&\xi ^s_1 = \bigg \{ x \in \mathbb {S}:\quad \mathcal {P}^3_N J(u^s)(x) = h, \quad \frac{\mathrm {d}}{\mathrm {d}x} \mathcal {P}^3_N J(u^s)(x) > 0 \bigg \}, \\&\xi ^s_2 = \bigg \{ x \in \mathbb {S}:\quad \mathcal {P}^3_N J(u^s)(x) = h, \quad \frac{\mathrm {d}}{\mathrm {d}x} \mathcal {P}^3_N J(u^s)(x) < 0 \bigg \}. \end{aligned}$$

We also point out that the computation stops if the two sets above are empty, whereupon, we set $\xi _1^s=\xi _2^s=0$.

The coarse time-stepper for bumps is then given by

32 $$\begin{aligned} \varPhi _\text {b} :\mathbb {S}^2 \rightarrow \mathbb {S}^2, \qquad \xi \mapsto ( \mathcal {R} \circ \mathcal {E}_T \circ \mathcal {L}_\text {b} ) (\xi ), \end{aligned}$$

where the dependence on parameter $\gamma$ has been omitted.

### Coarse time-stepper for travelling waves

In Sect. 7.1, we showed that the probability mass function, $\mu _\text {tw}(z)$, of a coarse travelling wave can be approximated numerically using the travelling wave of the deterministic model, by solving a simple set of eigenvalue problems. It is therefore natural to use $\mu _\text {tw}$ in the lifting procedure for the travelling wave. In analogy with what was done for the bump, our coarse variables $(\xi _1,\xi _2)$ are the boundaries of the activity set associated with the coarse wave, $X_\ge = [\xi _1, \xi _2]$. We then set

$$\begin{aligned} \mathcal {L}_\text {tw} :\mathbb {X}^2 \rightarrow \mathbb {U}^{N \times M}, \qquad (\xi _1,\xi _2)^T \mapsto \{ u^s(x) \}_s, \end{aligned}$$

where $\{u^s(x_i)\}_s$ are *M* independent samples of the probability mass functions $\mu _\text {tw}(x_i)$, with $c=(\xi _2-\xi _1)/3$. The restriction operator for travelling waves is the same as used for the bump. The coarse time-stepper for travelling waves, $\varPhi _\text {tw}$, is then obtained as in (32), with $\mathcal {L}_\text {b}$ replaced by $\mathcal {L}_\text {tw}$.

33 $$\begin{aligned} \varPhi _\text {tw} :\mathbb {S}^2 \rightarrow \mathbb {S}^2, \qquad \xi \mapsto ( \mathcal {R} \circ \mathcal {E}_T \circ \mathcal {L}_\text {tw} ) (\xi ). \end{aligned}$$

## Root finding and pseudo-arclength continuation

Once the coarse time-steppers, $\varPhi _\text {b}$ and $\varPhi _\text {tw}$, have been defined, it is possible to use Newton’s method and pseudo-arclength continuation to compute coarse states, continue them in one of the control parameters and assess their coarse linear stability. In this section, we will indicate dependence upon a single parameter $\gamma \in \mathbb {R}$, implying that this can be any of the control parameters in (8).

For bumps, we continue in $\gamma$ the nonlinear problem $F_\text {b}(\xi ;\gamma )=0$, where

34 $$\begin{aligned} F_\text {b} :\mathbb {S}^2 \times \mathbb {R}\rightarrow \mathbb {S}^2, \qquad \xi \mapsto \begin{bmatrix} \xi _1 \\ \xi _2 - \big ( \varPhi _\text {b}(\xi ; \gamma ) \big )_2 \end{bmatrix}. \end{aligned}$$

A vector $\xi$ such that $F_\text {b}(\xi ;\gamma )=0$ corresponds to a coarse bump with activity set $X_\ge = [0,\xi _2]$ and width $\xi _2$, occurring for the parameter value $\gamma$, that is, we eliminated the translation invariance associated with the problem by imposing $\xi _1=0$. In passing, we note that it is possible to hardwire the condition $\xi _1=0$ directly in $F_\text {b}$ and proceed to solve an equivalent 1-dimensional system. Here, we retain the 2-dimensional formulation with the explicit condition $\xi _1=0$, as this makes the exposition simpler.

During continuation, the explicitly unavailable Jacobians

$$\begin{aligned} D_\xi F_\text {b}(\xi ;\gamma ) = I - D_\xi \varPhi _\text {b}(\xi ; \gamma ), \qquad D_\gamma F_\text {b}(\xi ;\gamma ) = D_\gamma \varPhi _\text {b}(\xi ; \gamma ), \end{aligned}$$

are approximated using the first-order forward finite-difference formulas

$$\begin{aligned}&\varepsilon D_\xi \varPhi _\text {b}(\xi ; \gamma ) \widetilde{\xi } \approx \varPhi _\text {b}(\xi + \varepsilon \widetilde{\xi }; \gamma ) - \varPhi _\text {b}(\xi ; \gamma ), \\&\varepsilon D_\gamma \varPhi _\text {b}(\xi ; \gamma ) \widetilde{\gamma } \approx \varPhi _\text {b}(\xi ; \gamma + \varepsilon \widetilde{\gamma }) - \varPhi _\text {b}(\xi ; \gamma ). \end{aligned}$$

The finite difference formula for $D_\xi \varPhi _\text {b}$ also defines the Jacobian operator used to compute stability: for a given solution $\xi _*$ of (34), we study the associated eigenvalue problem

$$\begin{aligned} \lambda \xi = D_\xi \varPhi _\text {b}(\xi _*; \gamma ) \xi , \qquad \lambda \in \mathbb {C}, \quad \xi \in \mathbb {R}^2. \end{aligned}$$

For coarse travelling waves, we define

35 $$\begin{aligned} F_\text {tw} :\mathbb {S}^2 \times \mathbb {R}^2 \rightarrow \mathbb {S}^2, \qquad \begin{bmatrix} \xi \\ c \end{bmatrix} \mapsto \begin{bmatrix} \xi _1\\ \xi _2 -cT + \big (\varPhi _\text {b}\big (\xi ; \gamma \big ) \big )_2 \\ c - (\xi _2-\xi _1)/3 \end{bmatrix}. \end{aligned}$$

A solution $(\xi ,c)$ to the problem $F_\mathrm {tw}(\xi ,c;\gamma ) = 0$ corresponds to a coarse travelling wave with activity set $X_\ge = [0,\xi _2]$ and speed $\xi _2/3$, that is, we eliminated the translation invariance and imposed a speed *c* in accordance with the lifting procedure $\mathcal {L}_\mathrm {tw}$. As for the bump we can, in principle, solve an equivalent 1-dimensional coarse problem.

## Numerical results

**Fig. 14:**
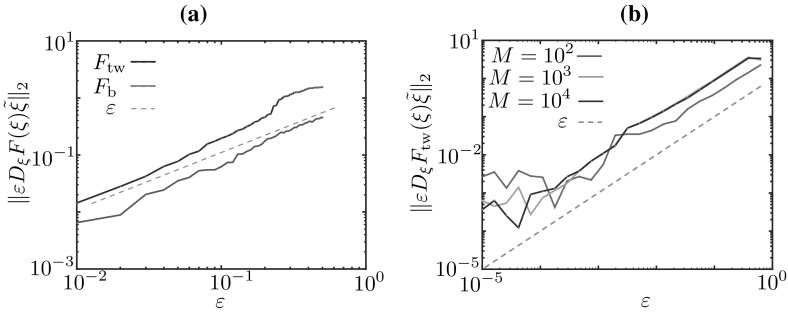
Jacobian-vector product norm as a function of $\varepsilon$. The approximated Jacobian-vector products $D_\xi F_\mathrm {b}(\xi ) \widetilde{\xi }$ and $D_\xi F_\mathrm {tw}(\xi ) \widetilde{\xi }$, are evaluated at a coarse bump and a coarse travelling wave $\xi$ in a randomly selected direction $\varepsilon \widetilde{\xi }$, where $\Vert \xi \Vert _2 = 1$. **a** A single realisation of the deterministic coarse-evolution maps is used in the test, showing that the norm of the Jacobian-vector product is an $\mathcal {O}(\varepsilon )$, as expected. Parameters: $p=1$, $\kappa =30$, $\beta \rightarrow \infty$ (Heaviside firing rate), $h=1$, $N=128$, $A_1 = 5.25$, $A_2=5$, $B_1=0.2$, $B_2=0.3$. **b** The experiment is repeated for a coarse travelling wave in the stochastic setting and for various values of *M*. Parameters as in (**a**), except $p=0.4$

We begin by testing the numerical properties of the coarse time-stepper, the Jacobian-vector products and the Newton solver used for our computations. In Fig. 14a, we evaluate the Jacobian- vector product of the coarse time stepper with $p=1$, $\beta \rightarrow \infty$ for bumps (waves) evaluated at a coarse bump (wave), in the direction $\varepsilon \widetilde{ \xi }$, where $0 < \varepsilon \ll 1$ and $\widetilde{\xi }$ is a random vector with norm 1. Since this coarse time stepper corresponds to the deterministic case, we expect the norm of the Jacobian-vector product to be an $O(\varepsilon )$, as confirmed by the numerical experiment. In Fig. 14b, we repeat the experiment in the stochastic setting ($p=0.4$), for the travelling wave case with various number of realisations. As expected, the norm of the Jacobian-vector action follows the $O(\varepsilon )$ curve for sufficiently large $\varepsilon$: the more realisations are employed, the more accurately the $O(\varepsilon )$ curve is followed.

**Fig. 15:**
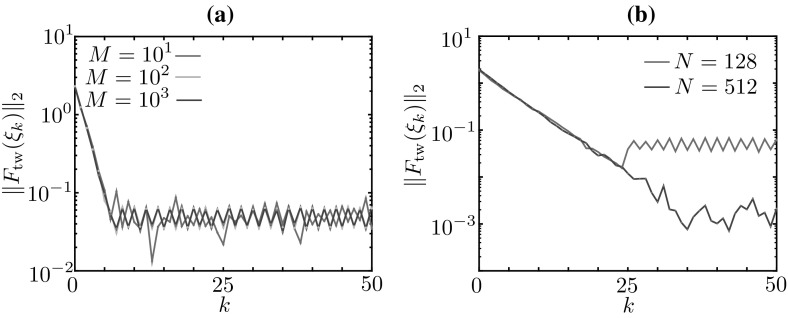
Convergence history of the damped Newton’s method applied to the coarse travelling wave problem. **a** The method converges linearly, and the achievable tolerance does not decrease when the number of realisations *M* is increased. **b** The achievable tolerance depends on the grid size, or, equivalently, on the number of neurons, *N*

We then proceed to verify directly the convergence history of the damped Newton solver. In Fig. 15a, we use a damping factor 0.5 and show the residual of the problem as a function of the number of iterations, showing that the method converges quickly to a solution. At first sight, it is surprising that the achievable tolerance of the problem does not change when the number of realisations increases. A second experiment, however, reported in Fig. 15b, shows that this behaviour is caused by the low system size: when we increase *N* from $2^7$ to $2^9$, the achievable tolerance decreases by one order of magnitude.

### Numerical Bifurcation analysis

Gong and Robinson (2012), and Qi and Gong (2015) found wandering spots and propagating ensembles using direct numerical simulations on the plane. Here, we perform a numerical bifurcation analysis with various control parameters for the structures found in Sect. 3 on a one-dimensional domain.

**Fig. 16:**
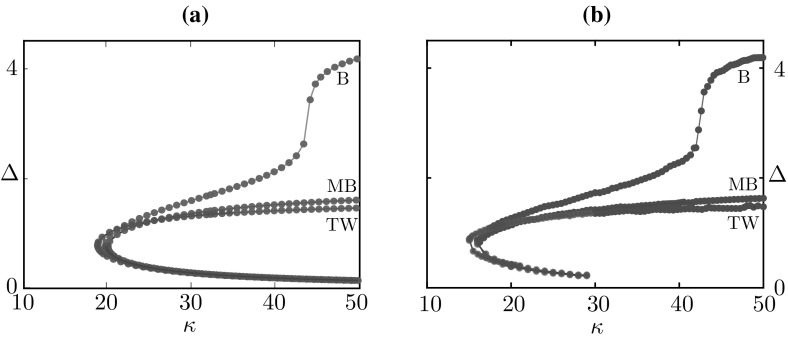
Bifurcation diagrams for bumps (B), multibumps (MB) and travelling waves (TW) using $\kappa$ as bifurcation parameter parameter. **a** Using the analytical results, we see that bump, multi-bump and travelling wave solutions coexist and are stable for sufficiently high $\kappa$ (see main text for details). **b** The solution branches found using the equation-free methods agree with the analytical results. Parameters as in Table 1 except $h=p=1.0$, $\beta \rightarrow \infty$

In Fig. 16a, we vary the primary control parameter $\kappa$, the gain of the convolution term, therefore, we study existence and stability of the bumps and the travelling pulse when the global coupling is varied. This continuation is performed for a bump, a multiple bump and a travelling pulse in the continuum deterministic model, using Eqs. (17), (20) and (23), respectively.

For sufficiently high $\kappa$, these states coexist and are stable in a large region of parameter space. We stress that spatially homogeneous mesoscopic states $J(x) \equiv J_*$, with $0 = J_*$ or $J_* > h$ are also supported by the model, but are not treated here. Interestingly, the three solution branches are disconnected, hence the bump analysed in this study does not derive from an instability of the trivial state. A narrow unstable bump $\varDelta \ll 1$ exists for arbitrarily large $\kappa$ (red branch); as $\kappa$ decreases, the branch stabilises at a saddle-node bifurcation. At $\kappa \approx 42$, the branch becomes steeper, the maximum of the bump changes concavity, developing a dimple. On an infinite domain, the branch displays an asymptote (not shown) as the bump widens indefinitely. On a finite domain, like the one reported in the figure, there is a maximum achievable width of the bump, due to boundary effects. The travelling wave is also initially unstable, but does not stabilise at the saddle node bifurcation. Instead, the wave becomes stable at $\kappa \approx 33$, confirming the numerical simulations reported in Fig. 9.

In Fig. 16b, we repeat the continuation for the same parameter values, but on a finite network, using the coarse time-steppers outlined in Sects. 8.1 and 8.2. The numerical procedure returns results in line with the continuum case, even at the presence of the noise induced by the finite size. The branches terminate for large $\kappa$ and low $\varDelta$: this can be explained by noting that, if $J(x) \equiv 0$, then the system attains the trivial resting state $u(x) \equiv 0$ immediately, as no neuron can fire; on a continuum network, $\varDelta$ can be arbitrarily small, hence the branch can be followed for arbitrarily large $\kappa$; on a discrete network, there is a minimal value of $\varDelta$ that can be represented with a finite grid.

**Fig. 17:**
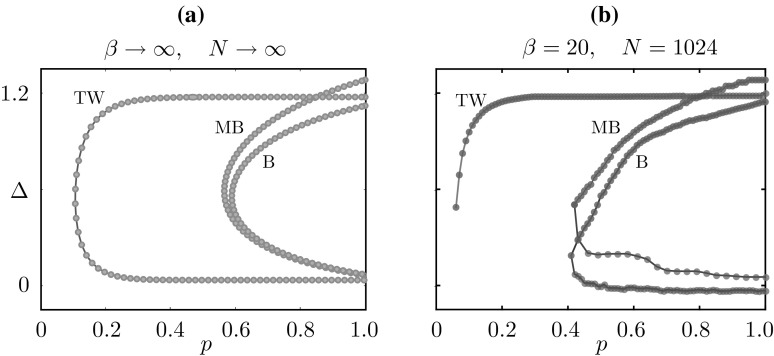
Bifurcation in the control parameter *p*. **a** Existence curves obtained analytically; we see that, below a critical value of *p*, only the travelling wave exists. **b** The solution branches found using the equation-free method agree qualitatively with the analytical results, and we can use the method to infer stability. For full details, please refer to the text. Parameters as in Table 1 except $\kappa =20.0,\,h=0.9$, with $\beta \rightarrow \infty$ for (**a**) and $\beta =20.0$ for (**b**)

**Fig. 18:**
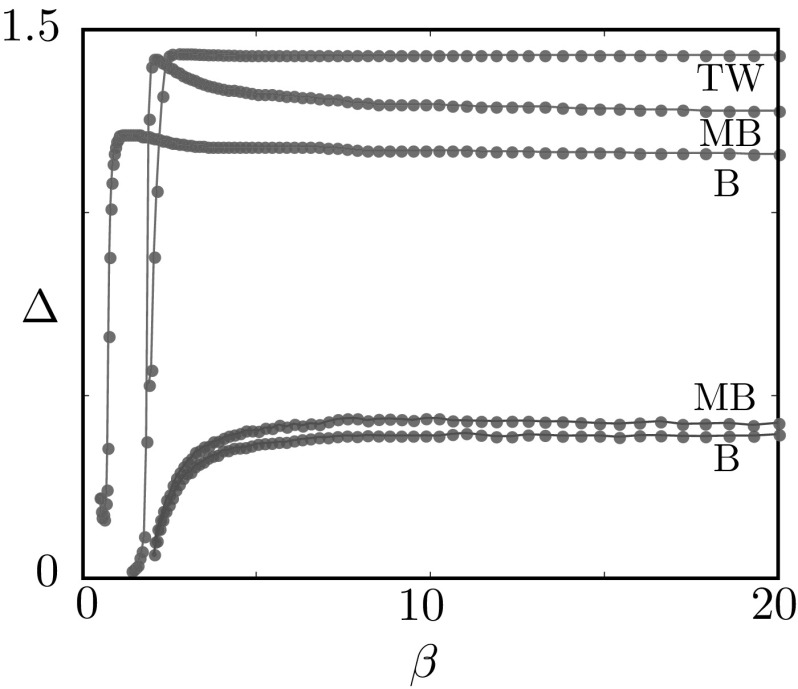
Bifurcation in the control parameter $\beta$. For a large range of values, we observe very little change in $\varDelta$ as $\beta$ is varied. Parameters as in Table 1 except $\kappa =40.0$, $h=0.9$, $p=1.0$. See the main text for full details

**Fig. 19:**
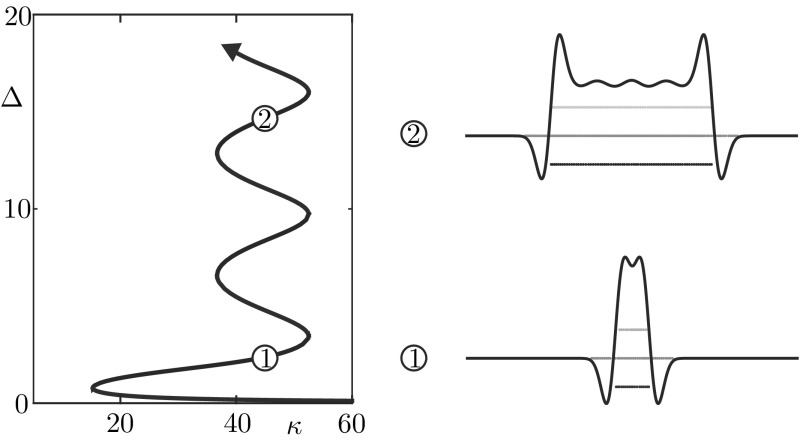
Bifurcation diagram for bumps in a heterogeneous network. To generate this figure, we replaced the coupling function with $\widetilde{W}(x,y)=W(x-y) (1+W_0 \cos (y/s))$, with $W_0=0.01,\,s=0.5$. We observe the snaking phenomenon in the approximate interval $\kappa \in [38,52]$. The branches moving upwards and to the right are stable, whereas those moving to the left are unstable. The images on the right, obtained via direct simulation, depict the solution profiles on the labelled part of the branches. We note the similarity of the mesoscopic profiles within the middle of the bump. The continuation was performed for the continumm, deterministic model with parameters are $\kappa =30$, $h=0.9$

We now consider continuation of solutions in the stochastic model. In Fig. 17, we vary the transition probability, *p*, from the refractory to quiescent state. In panel (a), we show analytical results, given by solving (28)–(29), whilst panel (b) shows results found using the equation-free method. We find qualitatively similar diagrams in both cases, though we note some quantitative differences, owing to the finite size of the network and the finiteness of $\beta$: at the presence of noise, the stationary solutions exist for a wider region of parameter space (compare the folds in Fig. 17a, b); a similar situation arises, is also valid for the travelling wave branches.

The analytical curves of Fig. 17a do not contain any stability information, which are instead available in the equation-free calculations of Fig. 17b, confirming that bump and multi-bump destabilise at a saddle-node bifurcation, whereas the travelling wave becomes unstable to perturbations in the wake, if *p* is too large. The lower branch of the travelling wave is present in the analytical results, but not in the numerical ones, as this branch is not captured by our lifting strategy: when we lift a travelling wave for very low values of $\varDelta$, we have that $J<h$ for all $x\in \mathbb {S}_N$ and the network attains the trivial state $u(x) \equiv 0$ in 1 or 2 time steps, thereby the coarse time stepper becomes ineffective, as the integration time *T* can not be reduced to 0.

Gong and Robinson (2012); Qi and Gong (2015) found that refractoriness is a key component to generating propagating activity in the network. The bifurcation diagram presented here confirm this, as we recognise 3 regimes: for high *p* (low refractory time) the system supports stationary bumps, as the wave is unstable; for intermediate *p*, travelling and stationary bumps coexist and are stable, while for low *p* (high refractory time) the system selects the travelling wave.

In Fig. 18, we perform the same computation now varying $\beta$, which governs the sensitivity of the transition from quiescence to spiking. Here, we see that the wave and both bump solutions are stable for a wide range of $\beta$ values and furthermore, that these states are largely insensitive to variations in this parameter, implying that the Heaviside limit is a good approximation for the network in this region of parameter space.

Finally, we apply the framework presented in the previous sections to study heterogeneous networks. We modulate the synaptic kernel using a harmonic function, as studied in Avitabile and Schmidt (2015) for a neural field. As in Avitabile and Schmidt (2015), the heterogeneity promotes the formation of a hierarchy of stable coexisting localised bumps, with varying width, arranged in a classical snaking bifurcation diagram as presented in Fig. 19. A detailed study of this bifurcation structure, while possible, is outside the scope of the present paper.

## Discussion

In this article, we have used a combination of analytical and numerical techniques to study pattern formation in a Markov chain neural network model. Whilst simple in nature, the model exhibits rich dynamical behaviour, which is often observed in more realistic neural networks. In particular, spatio-temporal patterns in the form of bumps have been linked to working memory (Funahashi et al. 1989; Colby et al. 1995; Goldman-Rakic 1995), whilst travelling waves are thought to be important for plasticity (Bennett 2015) and memory consolidation (Massimini et al. 2004; Rasch and Born 2013). Overall, our results reinforce the findings of Gong and Robinson 2012, namely that refractoriness is key to generating propagating activity: we have shown analytically and numerically that waves are supported by a combination of high gains in the synaptic input and moderate to long refractory times. For high gains and short refractory times, the network supports localised, meandering bumps of activity.

The analysis presented in this manuscript highlights the multiscale nature of the model by showing how evolution on a microscopic level gives rise to emergent behaviour at meso- and macroscopic levels. In particular, we established a link between descriptions of the model at multiple spatial scales: the identified coarse spatiotemporal patterns have typified and recognisable motifs at the microscopic level, which we exploit to compute macroscopic patterns and their stability.

To connect our micro- and macroscopic variables, we take advantage of interface approaches, which are typically applied to continuum networks. A notable exception is offered by Chow and Coombes (2006), who consider a network based upon the lighthouse model (Haken 2000a, b). In a similar vein to our approach, they show how analysis of the discrete network can be facilitated by considering a continuum approximation and derive threshold equations to define bump solutions. This analysis also highlights that perturbations to the microscopic state, specifically the phase arrangement within the bump, can alter the dynamics of the bump edges.

Chow and Coombes found that wandering bump solutions in the lighthouse model arise for sufficiently fast synaptic processing. This is congruent with our result that short refractory times in (8) elicit coherent bump states, since both refractory times and synaptic processing timescales affect the average firing rate of the neuron. However, bumps cease to exist in our model if the refractory times are too long, whereas the lighthouse model supports stationary bumps for slow synapses, which highlights the subtle differences between the roles of refractoriness and synaptic processing in neural networks. It should also be noted that the meandering observed, for instance, in Fig. 2 is due to noise, and that all bumps will tend to wander; on the other hand, the meandering described by Chow and Coombes arises from the deterministic dynamics of the lighthouse model, and it is triggered by a sufficently fast synaptic process. We also remark that, without modification, the lighthouse model does not support travelling wave solutions, and so we cannot make comparisons regarding these solutions.

Travelling waves and bumps have almost identical meso- and macroscopic profiles: if microscopic data were removed from Figs. 2a and 4a, the profiles and activity sets of these two patterns would be indistinguishable. We have shown that a disambiguation is however possible if the meso- and macroscopic descriptions take into account microscopic traits of the patterns: in the deterministic limit of the system, where mathematical analysis is possible, the microscopic structure is used in the partition sets of Propositions 1 and 2; in the stochastic setting with Heaviside firing rates and an infinite number of neurons, the microscopic structure is reflected in the approximate probability mass functions appearing in Sect. 7; in the full stochastic finite-size setting, where an analytical description is unavailable, the microscopic structure is hardwired in the lifting operators of the coarse time-steppers (Sect. 8).

An essential ingredient in our analysis is the dependence of the Markov chain transition probability matrix upon the global activity of the network, via the firing rate function *f*. Since this hypothesis is used to construct rate models as Markov processes (Bressloff 2010), our lifting strategy could be used in equation-free schemes for more general large-dimensional neural networks. An apparent limitation of the procedure presented here is its inability to lift strongly unstable patterns with low activity, as pointed out in Sect. 10. This limitation, however, seems to be specific to the model studied here: when $\varDelta \rightarrow 0$, bumps destabilise with transients that are too short to be captured by the coarse time-stepper.

A possible remedy would be to represent the pattern via a low-dimensional, spatially-extended, spectral discretisation of the mesoscopic profile (see Laing (2006)), which would allow us to represent the synaptic activity below the threshold *h*. This would lead to a larger-dimensional coarse system, in which noise would pollute the Jacobian-vector evaluation and the convergence of the Newton method. Variance-reduction techniques (Rousset and Samaey 2013) have been recently proposed for equation-free methods in the context of agent-based models (Avitabile et al. 2014), and we aim to adapt them to large neural networks in subsequent publications.

## References

[CR1] Amari Si (1975). Homogeneous nets of neuron-like elements. Biol Cybern.

[CR2] Amari Si (1977). Dynamics of pattern formation in lateral-inhibition type neural fields. Biol Cybern.

[CR3] Avitabile D, Hoyle R, Samaey G (2014). Noise reduction in coarse bifurcation analysis of stochastic agent-based models: an example of consumer lock-in. SIAM J Appl Dyn Syst.

[CR4] Avitabile D, Schmidt H (2015). Snakes and ladders in an inhomogeneous neural field model. Physica D.

[CR5] Baladron J, Fasoli D, Faugeras O, Touboul J (2012) Mean-field description and propagation of chaos in networks of Hodgkin-Huxley and FitzHugh-Nagumo neurons. J Math Neurosci 2(10):1–50 10.1186/2190-8567-2-10 PMC3497713 22657695

[CR6] Bennett JEM (2015). Refinement and pattern formation in neural circuits by the interaction of traveling waves with spike-timing dependent plasticity. PLoS Comput Biol.

[CR7] Brackley CA, Turner MS (2007). Random fluctuations of the firing rate function in a continuum neural field model. Phys Rev E.

[CR8] Braitenberg V, Schüz A (1998). Cortex: statistics and geometry of neuronal connectivity.

[CR9] Bressloff PC (2009). Stochastic neural field theory and the system-size expansion. SIAM J Appl Math.

[CR10] Bressloff PC (2010). Stochastic neural field theory and the system-size expansion. SIAM J Appl Math.

[CR11] Bressloff PC (2012). Spatiotemporal dynamics of continuum neural fields. J Phys A Math Theor.

[CR12] Bressloff PC (2014) Waves in neural media. Lecture notes on mathematical modelling in the life sciences. Springer, New York

[CR13] Bressloff PC, Cowan JD, Golubitsky M, Thomas PJ (2001). Scalar and pseudoscalar bifurcations motivated by pattern formation on the visual cortex. Nonlinearity.

[CR14] Bressloff PC, Kilpatrick ZP (2011). Two-dimensional bumps in piecewise smooth neural fields with synaptic depression. SIAM J Appl Math.

[CR15] Bressloff PC, Webber MA (2012). Front propagation in stochastic neural fields. SIAM J Appl Dyn Syst.

[CR16] Cai D, Tao L, Shelley M, McLaughlin DW (2004). An effective kinetic representation of fluctuation-driven neuronal networks with application to simple and complex cells in visual cortex. Proc Nat Acad Sci.

[CR17] Chow C, Coombes S (2006). Existence and wandering of bumps in a spiking neural network model. SIAM J Appl Dyn Syst.

[CR18] Colby C, Duhamel J, Goldberg M (1995). Oculocentric spatial representation in parietal cortex. Cereb Cortex.

[CR19] Coombes S, Owen MR (2004). Evans functions for integral neural field equations with heaviside firing rate function. SIAM J Appl Dyn Syst.

[CR20] Coombes S, Schmidt H, Avitabile D (2014) Neural field theory, chap. Spots: breathing, drifting and scattering in a neural field model. Springer, Berlin, pp 187–211

[CR21] Coombes S, Schmidt H, Bojak I (2012). Interface dynamics in planar neural field models. J Math Neurosci.

[CR22] Einevoll GT, Kayser C, Logothetis NK, Panzeri S (2013). Modelling and analysis of local field potentials for studying the function of cortical circuits. Nat Rev Neurosci.

[CR23] Ermentrout GB (1998). Neural networks as spatio-temporal pattern-forming systems. Rep Prog Phys.

[CR24] Ermentrout GB, Cowan JD (1979). A mathematical theory of visual hallucination patterns. Biol Cybern.

[CR25] Ermentrout GB, McLeod JB (1993). Existence and uniqueness of travelling waves for a neural network. Proc R Soc Edinb Sect A Math.

[CR26] Ermentrout GB, Terman DH (2010). Mathematical foundations of neuroscience.

[CR27] Fairhall A, Sompolinsky H (2014) Editorial overview: theoretical and computational neuroscience. Curr Opinion Neurobiol 25:v–viii 10.1016/j.conb.2014.02.010 24656299

[CR28] Faugeras O, Touboul J, Cessac B (2009). A constructive mean-field analysis of multi-population neural networks with random synaptic weights and stochastic inputs. Front Comput Neurosci.

[CR29] Faye G, Rankin J, Lloyd DJB (2013). Localized radial bumps of a neural field equation on the Euclidean plane and the Poincaré disk. Nonlinearity.

[CR30] Folias SE, Bressloff PC (2004). Breathing pulses in an excitatory neural network. SIAM J Appl Dyn Syst.

[CR31] Folias SE, Bressloff PC (2005). Breathers in two-dimensional neural media. Phys Rev Lett.

[CR32] Folias SE, Ermentrout GB (2012). Bifurcations of sationary solutions in an interacting pair of E-I neural fields. SIAM J Appl Dyn Syst.

[CR33] Funahashi S, Bruce C, Goldman-Rakic P (1989). Mnemonic coding of visual space in the monkey’s dorsolateral prefrontal cortex. J Neurophysiol.

[CR34] Goldman-Rakic P (1995). Cellular basis of working memory. Neuron.

[CR35] Golomb D, Ermentrout GB (1999). Continuous and lurching traveling pulses in neuronal networks with delay and spatially decaying connectivity. Proc Nat Acad Sci.

[CR36] Gong P, Robinson PA (2012) Dynamic pattern formation and collisions in networks of excitable elements. Phys Rev E 85(5):055,101(R) 10.1103/PhysRevE.85.055101 23004809

[CR37] Haskell E, Nykamp DQ, Tranchina D (2001). A population density method for large-scale modeling of neuronal networks with realistic synaptic kinetics. Neurocomputing.

[CR38] Haken H (2000). Phase locking in the lighthouse model of a neural net with several delay times. Prog Theor Phys.

[CR39] Haken H (2000). Quasi-discrete dynamics of a neural net: The lighthouse model. Discret Dyn Nat oc.

[CR40] Hutt A, Longtin A, Schimansky-Geier L (2008). Additive noise-induced turing transitions in spatial systems with application to neural fields and the swift-hohenberg equation. Physica D.

[CR41] Izhikevich EM (2007) Dynamical systems in neuroscience: The Geometry of Excitability and Bursting. MIT Press

[CR42] Izhikevich EM, Edelman GM (2008). Large-scale model of mammalian thalamocortical systems. Proc Nat Acad Sci.

[CR43] Jirsa VK, Haken H (1997). A derivation of a macroscopic field theory of the brain from the quasi-microscopic neural dynamics. Physica D.

[CR44] Kevrekidis IG, Gear CW, Hyman JM, Kevrekidis PG, Runborg O, Theodoropoulos C (2003). Equation-free, coarse-grained multiscale computation: enabling microscopic simulators to perform system-level tasks. Commun Math Sci.

[CR45] Kevrekidis IG, Samaey G (2009). Equation-free multiscale computation: algorithms and applications. Annu Rev Phys Chem.

[CR46] Kilpatrick ZP, Bressloff PC (2010). Stability of bumps in piecewise smooth neural fields with nonlinear adaptation. Physica D.

[CR47] Kilpatrick ZP, Ermentrout GB (2013). Wandering bumps in stochastic neural fields. SIAM J Appl Dyn Syst.

[CR48] Kuehn C, Riedler M (2014). Large deviations for nonlocal stochastic neural fields. J Math Neurosci.

[CR49] Laing CR (2005). Spiral waves in nonlocal equations. SIAM J Appl Dyn Syst.

[CR50] Laing CR (2006). On the application of ‘equation-free modelling’ to neural systems. J Comput Neurosci.

[CR51] Laing CR, Frewen T, Kevrekidis IG (2010). Reduced models for binocular rivalry. J Comput Neurosci.

[CR52] Laing CR, Frewen TA, Kevrekidis IG (2007). Coarse-grained dynamics of an activity bump in a neural field model. Nonlinearity.

[CR53] Laing CR, Kevrekidis IG (2015). Equation-free analysis of spike-timing-dependent plasticity. Biol Cybern.

[CR54] Laing CR, Troy WC (2003). PDE methods for nonlocal models. SIAM J Appl Dyn Syst.

[CR55] Laing CR, Troy WC, Gutkin B, Ermentrout GB (2002). Multiple bumps in a neuronal model of working memory. SIAM J Appl Math.

[CR56] Ly C, Tranchina D (2007). Critical analysis of dimension reduction by a moment closure method in a population density approach to neural network modeling. Neural Comput.

[CR57] Markram H, Muller E, Ramaswamy S, Reimann MW, Abdellah M, Sanchez CA, Ailamaki A, Alonso-Nanclares L, Antille N, Arsever S, Kahou GAA, Berger TK, Bilgili A, Buncic N, Chalimourda A, Chindemi G, Courcol JD, Delalondre F, Delattre V, Druckmann S, Dumusc R, Dynes J, Eilemann S, Gal E, Gevaert ME, Ghobril JP, Gidon A, Graham JW, Gupta A, Haenel V, Hay E, Heinis T, Hernando JB, Hines M, Kanari L, Keller D, Kenyon J, Khazen G, Kim Y, King JG, Kisvarday Z, Kumbhar P, Lasserre S, Le Bé JV, Magalhães BRC, Merchán-Pérez A, Meystre J, Morrice BR, Muller J, Muñoz-Céspedes A, Muralidhar S, Muthurasa K, Nachbaur D, Newton TH, Nolte M, Ovcharenko A, Palacios J, Pastor L, Perin R, Ranjan R, Riachi I, Rodríguez JR, Riquelme JL, Rössert C, Sfyrakis K, Shi Y, Shillcock JC, Silberberg G, Silva R, Tauheed F, Telefont M, Toledo-Rodriguez M, Tränkler T, Van Geit W, Díaz JV, Walker R, Wang Y, Zaninetta SM, Defelipe J, Hill SL, Segev I, Schürmann F (2015). Reconstruction and simulation of neocortical microcircuitry. Cell.

[CR58] Massimini M, Huber R, Ferrarelli F, Hill S, Tononi G (2004). The sleep slow oscillation as a traveling wave. J Neurosci.

[CR59] Nunez PL, Srinivasan R (2006) Electric fields of the brain: the neurophysics of EEG, 2nd edn. Oxford University Press

[CR60] Omurtag A, Knight BW, Sirovich L (2000). On the simulation of large populations of neurons. J Comput Neurosci.

[CR61] Osan R, Ermentrout B (2001). Two dimensional synaptically generated traveling waves in a theta-neuron neural network. Neurocomputing.

[CR62] Owen MR, Laing CR, Coombes S (2007). Bumps and rings in a two-dimensional neural field: splitting and rotational instabilities. New J Phys.

[CR63] Qi Y, Gong P (2015). Dynamic patterns in a two-dimensional neural field with refractoriness. Phys Rev E.

[CR64] Rankin J, Avitabile D, Baladron J, Faye G, Lloyd DJB (2014). Continuation of localized coherent structures in nonlocal neural field equations. SIAM J Sci Comput.

[CR65] Rasch B, Born J (2013). About sleep’s role in memory. Physiol Rev.

[CR66] Rousset M, Samaey G (2013). Simulating individual-based models of bacterial chemotaxis with asymptotic variance reduction. Math Models Methods ApplSci.

[CR67] Spiliotis KG, Siettos CI (2011). A timestepper-based approach for the coarse-grained analysis of microscopic neuronal simulators on networks: Bifurcation and rare-events micro-to macro-computations. Neurocomputing.

[CR68] Spiliotis KG, Siettos CI (2012). Multiscale computations on neural networks: from the individual neuron interactions to the macroscopic-level analysis. Int J Bifurcation Chaos.

[CR69] Tuckerman LS, Barkley D (2000) Bifurcation analysis for timesteppers. In: Numerical methods for Bifurcations of dynamical equilibria. SIAM, New York, pp 453–466

[CR70] van den Heuvel MP, Hulshoff Pol HE (2010). Exploring the brain network: a review on resting-state fMRI functional connectivity. Eur Neuropsychopharmacol.

[CR71] Weinan W, Engquist B (2003). The heterogeneous multiscale methods. Commun Math Sci.

[CR72] Weinan E, Engquist B, Li X, Ren W, Vanden-Eijden E (2007). Heterogeneous multiscale method: a review. Commun Comput Phys.

[CR73] Wasylenko TM, Cisternas JE, Laing CR, Kevrekidis IG (2010). Bifurcations of lurching waves in a thalamic neuronal network. Biol Cybern.

[CR74] Werner H, Richter T (2001). Circular stationary solutions in two-dimensional neural fields. Biol Cybern.

[CR75] Wilson HR, Cowan JD (1972). Excitatory and inhibitory interactions in localized populations of model neurons. Biophys J.

[CR76] Wilson HR, Cowan JD (1973). A mathematical theory of the functional dynamics of cortical and thalamic nervous tissue. Biol Cybern.

[CR77] Yuste R (2015). From the neuron doctrine to neural networks. Nat Rev Neurosci.

